# Methylation-based smoking signatures in blood and tissue samples for the prediction of self-reported smoking status and mortality in patients with colorectal cancer

**DOI:** 10.1186/s13148-025-01918-9

**Published:** 2025-07-03

**Authors:** Tanwei Yuan, Katrin E. Tagscherer, Wilfried Roth, Melanie Bewerunge-Hudler, Alexander Brobeil, Matthias Kloor, Hendrik Bläker, Hermann Brenner, Michael Hoffmeister

**Affiliations:** 1 Division of Clinical Epidemiology and Aging Research, Molecular Pathological Epidemiology Group, German Cancer Research Center (DKFZ), Im Neuenheimer Feld 581, 69120 Heidelberg, Germany; 2 Medical Faculty Heidelberg, Heidelberg University, Heidelberg, Germany; 3 Institute of Pathology, University Medical Center Mainz, Mainz, Germany; 4 Institute of Pathology, Heidelberg University Hospital, Heidelberg, Germany; 5 Microarray Core Facility, German Cancer Research Center (DKFZ), Heidelberg, Germany; 6 Institute of Pathology, University of Leipzig Medical Center, Leipzig, Germany; 7 German Cancer Consortium (DKTK), German Cancer Research Center (DKFZ), Heidelberg, Germany

**Keywords:** Smoking, DNA methylation biomarkers, Colorectal cancer, Prognosis

## Abstract

**Background:**

Smoking is a well-established risk factor for colorectal cancer (CRC) development. However, the reliability of DNA methylation-based smoking signatures in predicting smoking status and their prognostic value in CRC remain unclear, particularly across different biological sample types.

**Results:**

Five previously validated methylation-based smoking signatures were analyzed in 2237 CRC patients with blood-derived DNA and 2273 patients with tumor tissue-derived DNA. Blood-derived signatures showed strong correlations with self-reported smoking status, effectively differentiating current smokers from never smokers (all *p* < 0.0001), with excellent discriminative ability (median area under the receiver operating characteristic curve: 0.94). In contrast, tumor tissue-derived signatures exhibited much weaker associations with smoking status. Among non-metastatic CRC patients, blood-derived methylation signatures were significantly associated with increased risks of all-cause and non-CRC-related mortality, but not with CRC-specific mortality. Conversely, two tumor tissue-derived signatures demonstrated stronger associations with CRC-specific mortality compared to blood-derived signatures.

**Conclusions:**

Blood-derived methylation-based smoking signatures are robust indicators for smoking exposure and are associated with increased mortality risk among non-metastatic CRC patients. When applied to tumor tissue, signatures showed stronger associations with CRC-specific mortality.

**Supplementary Information:**

The online version contains supplementary material available at 10.1186/s13148-025-01918-9.

## Background

Colorectal cancer (CRC) remains one of the most common cancers and a leading cause of cancer-related deaths worldwide [1, 2]. Smoking is a well-established risk factor for CRC, influencing both its incidence and prognosis [3–5]. According to several large patient cohort studies and meta-analyses, smoking is strongly associated with risk of mortality among patients with CRC, especially among stage I–III patients [3, 6–8]. Accurate assessment of smoking status is crucial for understanding its impact on clinical outcomes and tailoring personalized monitoring strategies.

Traditionally, smoking status has been assessed through self-reporting [9, 10], which, while widely used, is subject to bias and inaccuracies. Misreporting or underreporting smoking behaviors and smoking amount can lead to misclassification of smoking-related risks [9, 10]. In response to these limitations, several methylation-based smoking signatures derived from blood samples have been proposed to quantify smoking exposure [11–15]. These epigenetic markers offer the potential to capture cumulative exposures to smoking, providing a more comprehensive picture of an individual’s smoking history [12]. Moreover, they can reflect the biological impact of smoking more accurately, accounting for individual variations in response to smoking exposure [12].

Still, the utility of methylation-based smoking signatures in predicting self-reported smoking status and their potential impact on survival outcomes in patients with CRC is yet to be fully explored. Moreover, it is unclear whether these methylation-based scores, originally identified in blood samples, can maintain their relevance when applied to tissue samples. This study aims to investigate the predictive value of methylation-based smoking signatures in both blood and tumor tissue samples among patients with colorectal cancer. By comparing these epigenetic markers with self-reported smoking data, we seek to evaluate their reliability as indicators of smoking exposure and their associations with patient survival.

## Methods

### Study cohort

We conducted and reported this study according to The Strengthening the Reporting of Observational Studies in Epidemiology (STROBE) Statement [16]. The German Darmkrebs: Chancen der Verhütung durch Screening (DACHS, English name "Colorectal cancer: chances for prevention through screening”) study is a large population-based case–control and patient cohort study on CRC conducted in the Rhine-Neckar region in the southwest of Germany from 2003 to 2021. Details of this study have been previously described [17, 18]. Briefly, German-speaking patients aged over 30 with a first histologically confirmed primary CRC diagnosis, physically and mentally capable of a one-hour interview, were recruited from 22 hospitals in the study region.

### Data collection

At baseline, trained interviewers collected data on sociodemographic characteristics, lifestyle, medical history, and disease symptoms through a standardized interview. Tumor characteristics and disease stage (TNM 6th edition) were obtained from medical records [19]. Smoking history before CRC diagnosis was recorded, classifying patients as current, former (quitted at least two years before diagnosis), and never smokers, and quantifying lifetime cumulative exposure by pack-years [20].

Peripheral blood samples were collected post-interview and stored at − 80 °C. Methylation assessment of whole-blood DNA was performed in a subsample of 2242 patients diagnosed with CRC between 2003 and 2010 using the Infinium MethylationEPIC BeadChip Kit (Illumina, San Diego, CA, USA), covering over 850,000 CpG sites. Molecular analyses of tumor tissue were conducted using DNA extracted from formalin-fixed, paraffin-embedded samples. Genome-wide methylation analysis was performed on tissue DNA in a subsample of 2316 patients diagnosed with CRC between 2003 and 2013 using the Illumina Human Methylation 450 BeadChip, covering over 485,000 CpG sites.

Standardized information on CRC therapy, comorbidities, and recurrence was obtained from physicians during follow-up visits at 3, 5, and 10 years after diagnosis. Vital status, date, and cause of death were collected from local population registries and public health authorities. Only patients with complete smoking and DNA methylation data were included in this study (*N* = 2237 for blood samples, *N* = 2273 for tumor tissue samples).

### Calculation of methylation-based smoking signatures

**Fig. 1:**
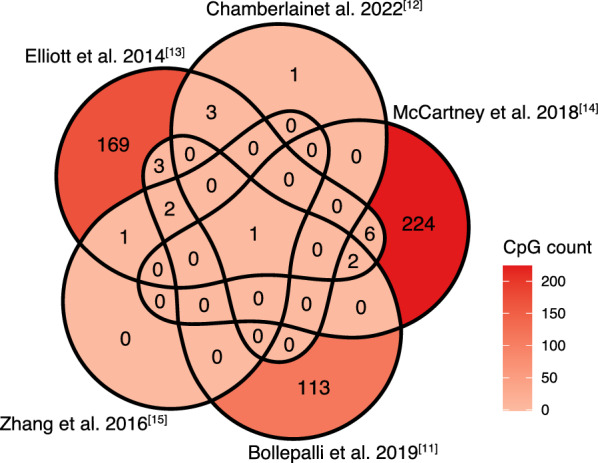
Overlapping CpGs of proposed blood methylation-based smoking signatures

We selected five previously validated blood methylation-based smoking signatures (Fig. 1) [11–15]. Four of these used methylation scores calculated as a linear combination of smoking-associated CpGs [12–15], while one predicted smoking status (current, former, and never) based on 121 CpGs and sex [11]. These signatures contained between 4 and 233 CpGs, with one (cg05575921) common to all and four (cg05951221, cg06126421, cg06644428, cg21566642) shared by three. Most CpGs were unique to each signature (Fig. 1).

The raw DNA methylation data file from the iScan array scanner was processed using the ‘minfi’ R package. Missing CpG data were imputed with nearest averaging multiple imputation. For each signature, we used the same normalization methods as those used in the original studies that developed the respective score (Supplement eTable 1) [11]. We did not apply additional CpG filtering beyond the original signature definitions. Instead, we extracted the CpG sites corresponding to each signature, based on their availability in our dataset. No further feature selection or filtering was performed.

We evaluated the coverage of CpG sites for each signature on both methylation platforms (450k and 850k arrays) and summarized the proportion of available CpGs per signature (Supplement eTable 1). All CpG sites required for the five signatures were available on the 450k array used for tissue sample. For the 850k array used for blood sample, CpG coverage was complete for two signatures (100%) [12, 14], while two others showed high but incomplete coverage, with 93% (174/187 CpGs) [13] and 91% (111/121 CpGs) [11] available, respectively. For the 4-CpG signature by Zhang et al. [15], only two CpGs (50%) were present on the 850k array.

The ‘EpiSmokEr’ R package [11] was used to predict smoking status and two smoking scores [13, 15]. The other two scores [12, 14] were calculated using linear equations provided by the original publications. The distributions of the five smoking signatures, as well as the individual CpG sites included in these signatures, were compared between blood and tumor tissue samples.

### Statistical analysis

All analyses were stratified by the source of DNA methylation data. Primary analyses focused on blood (*N* = 2237) and tumor tissue (*N* = 2273) samples, while secondary analyses used data derived from 264 adjacent normal tissue samples. Patient characteristics were summarized using descriptive statistics.

Smoking score distributions across categories of self-reported smoking status were compared using violin plots and Tukey's honestly significant difference test when normality assumptions were met, or the Wilcoxon rank-sum test otherwise. The Bonferroni method was used to account for multiple comparisons. Associations between the smoking scores and pack-years were visualized with scatter plots and measured by the Spearman correlation metrics. We additionally compared the distribution of self-reported smoking status and methylation-based smoking signatures by TNM stage.

Multivariable binary logistic regression was used to quantify the relationship between each epigenetic smoking signature and self-reported smoking status, adjusting for age, sex, body mass index (BMI) 5–14 years earlier, alcohol consumption, physical activity, use of nonsteroidal anti-inflammatory drugs, hormone replacement therapy, and prior large bowel endoscopy. McFadden’s pseudo-R^2^ was used to assess the explanatory power of each signature. The area under the receiver operating characteristic curve (AUROC) with 95% confidence intervals (2000 stratified bootstrap replicates) was used to measure the discriminative power. To account for the potential confounding effect of treatment-related epigenetic changes, we performed additional sensitivity analyses restricting to patients who had not received chemotherapy or radiotherapy.

Cox proportional hazards models were used to evaluate the association between smoking exposure (self-reported and methylation-predicted) and all-cause mortality. The proportional hazards assumption was checked using the scaled Schoenfeld residuals. The median follow-up time was computed using the reverse Kaplan–Meier method. Delayed-entry Cox models were used to address time differences between CRC diagnosis and sample collection [21]. Cause-specific Cox models were applied for CRC-specific and other mortality, with death from other causes considered as a competing risk. Models were adjusted for age, sex, body mass index (BMI) at CRC diagnosis, physical activity, alcohol consumption, TNM stage, tumor location, and treatment with chemotherapy or radiotherapy. The primary survival analysis included stage I-III CRC patients (blood, *N* = 1911; tumor tissue, *N* = 1959) [6, 7], with stage IV patients analyzed separately. Interaction terms between each methylation signature, age, and sex were tested for effect modification, followed by stratified analyses if required. Sensitivity analysis was performed among overlapping patients with both blood and tissue samples available.

Regression results for methylation-derived scores were standardized, with one-unit increases corresponding to one standard deviation. Participants with missing values for covariables, which were all very rare (below 2%), were excluded from regression analyses. Statistical significance was set as *p* value < 0.05 in two-sided testing. All analyses were performed using R version 4.2.0.

### Patient and public invlvement

Patients or the public were not involved in the designing, or conduct, or reporting or dissemination plans of our research.

## Results

### Characteristics of the study cohort

The characteristics of patients with blood methylation data were comparable to those with tumor tissue methylation data (Table 1).

**Table 1:** Characteristics of the analyzed study populations

Characteristics	Blood sample	Tumor tissue sample	*P* value
	(*N* = 2237)^1^	(*N* = 2273)^1^	
Median age (IQR)	69 (62, 77)	70 (62, 77)	0.573
Sex			0.807
Female	922 (41.2)	946 (41.6)	
Male	1315 (58.8)	1327 (58.4)	
Smoking status			0.937
Never	1037 (46.4)	1048 (46.1)	
Former	858 (38.4)	883 (38.8)	
Current	342 (15.3)	342 (15.0)	
Pack-years of smoking^2^			0.917
1–10	391 (33.6)	411 (33.6)	
10–19	282 (23.5)	291 (23.8)	
20–29	210 (17.5)	197 (16.1)	
> 30	308 (25.7)	317 (25.9)	
Missing	9	9	
BMI (kg/m^2^) 5–14 years earlier			0.930
Underweight (< 18.5)	12 (0.5)	11 (0.5)	
Normal (18.5–25)	691 (30.9)	717 (31.5)	
Overweight (25–30)	1059 (48.1)	1069 (47.7)	
Obese (≥ 30)	440 (20.0)	446 (19.9)	
Missing	35	30	
BMI (kg/m^2^) at diagnosis			0.864
Underweight (< 18.5)	50 (2.2)	49 (2.2)	
Normal (18.5–25)	822 (36.7)	846 (37.2)	
Overweight (25–30)	931 (41.8)	953 (42.1)	
Obese (≥ 30)	424 (19.0)	417 (18.4)	
Missing	10	8	
Alcohol drinking (days/week)			0.952
Never drinker (0)	674 (30.2)	684 (30.2)	
Light drinker (1–2)	521 (23.4)	538 (23.7)	
Heavy drinker (≥ 3)	1036 (46.4)	1045 (46.1)	
Missing	6	6	
Physical activity (lifetime average MET-hours/week)			0.345
Median (IQR)	191.1 (129.9, 277.3)	189.8 (129.4, 269.1)	
Missing	39	31	
Use of nonsteroidal anti-inflammatory drugs	548 (24.5)	591 (26.0)	0.259
Use of hormone replacement therapy^3^	270 (29.3)	275 (29.1)	1.000
Missing	4	2	
Prior large bowel endoscopy	500 (22.4)	526 (23.2)	0.550
Missing	1	1	
TNM stage			
I	403 (18.1)	413 (18.2)	0.990
II	767 (34.5)	784 (34.6)	
III	741 (33.3)	745 (32.9)	
IV	314 (14.1)	325 (14.3)	
Missing	12	6	
Tumor location			
Distal colon	610 (27.3)	629 (27.7)	0.523
Proximal colon	808 (36.1)	848 (37.3)	
Rectum	818 (36.6)	795 (35.0)	
Missing	1	1	
Treatment with chemotherapy/radiotherapy	1084 (48.5)	1062 (46.8)	0.273
Missing	3	6	

*IQR* Interquartile range, *BMI* Body mass index, *MET* Metabolic equivalent of task, *TNM* Tumor, lymph node, and metastasis staging system. ^1^The two sample groups contain 2008 overlapping patients. ^2^Only among former or current smokers. ^3^Only among female participants

Both cohorts overlapped largely (2008 same patients), with very comparable patient characteristics (all *p* value > 0.1) sample size (blood: 2237; tumor tissue: 2273), median ages (blood: 69 years; tumor tissue: 70 years), percentages of male (blood: 58.8%, tumor: 58.4%), and percentages of stage IV CRC patients (blood sample: 14.0%, tumor tissue: 14.3%). Approximately 15% of patients were current smokers, and 46% had never smoked. The median follow-up time for stage I-III patients was 10.6 years (IQR 10.2–13.5) for those with a blood sample and 10.5 years (IQR 10.1–12.3) for those with a tumor tissue sample, with 10-year mortality rates of 43.6% and 50.6%, respectively.

### Distribution of methylation-based smoking signatures

All five smoking scores, and nearly all constituent CpG sites, showed significantly different distributions between the two tissue types (Supplement eTable 2). With the exception of the score developed by Zhang et al. [15], tumor tissue-derived scores were significantly higher than those derived from blood samples. In addition, the smoking status predicted using tumor tissue-derived scores classified a substantially higher proportion of individuals as current smokers (74.1%) compared to the blood-derived predictor (29.8%). Stage IV patients had a higher proportion of self-reported current smokers and higher blood methylation-based smoking score developed by Zhang et al. (Supplement eFigure 1) [15].

### Associations between epigenetic smoking signatures and self-reported smoking status

**Fig. 2:**
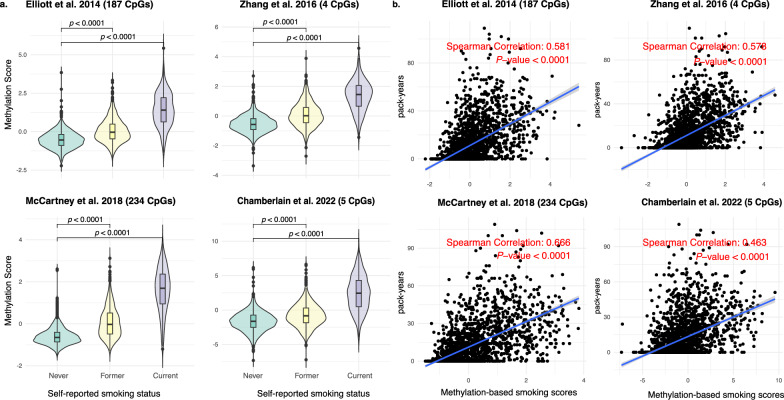
**A** Distribution of blood-derived methylation-based smoking scores across self-reported smoking groups; **B** Scatterplots showing the association between blood-derived methylation-based smoking scores and self-reported pack-years

**Fig. 3:**
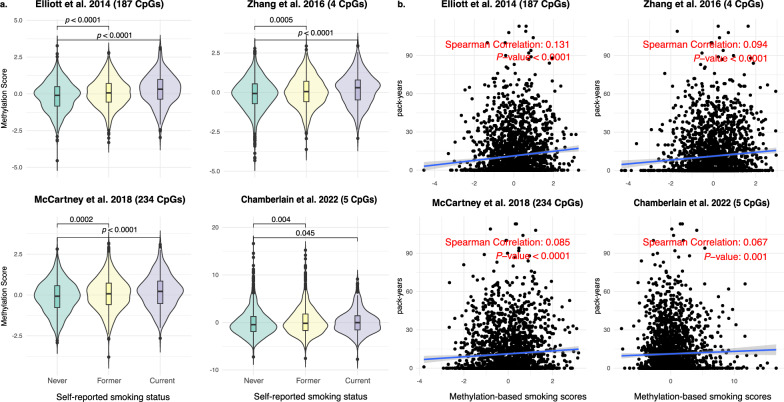
**A** Distribution of tumor tissue-derived methylation-based smoking scores across self-reported smoking groups; **B** Scatterplots showing the association between tumor tissue-derived methylation-based smoking scores and self-reported pack-years

In patients with blood DNA methylation data, all four methylation-based smoking scores increased progressively across never, former, and current smokers (Fig. 2A, *p* < 0.0001). A positive correlation was found between these scores and pack-years (median Spearman correlation: 0.58, *p* < 0.0001; Fig. 2B), with the McCartney et al. [14] score showing the strongest correlation (0.67). In tumor DNA methylation data, a similar but weaker pattern was observed across smoking groups (Fig. 3A), with very weak correlations with pack-years (median Spearman correlation: 0.09; Fig. 3B).

All five blood-derived DNA methylation-based signatures were strongly associated with smoking status in the adjusted model (all *p* < 0.0001; Table 2), especially for current versus never smokers (median McFadden’s pseudo-*R*^2^: 0.56, range 0.37–0.70). Discrimination between current and never smokers was almost perfect (AUROC median: 0.94; range 0.92–0.96), followed by current versus former smoking (0.85, 0.75–0.88). The strongest associations were found for the two signatures developed by McCartney et al. [14] and Bollepalli et al. [11], respectively.

**Table 2:** Associations between methylation-based smoking scores and self-reported smoking status

Methylation score	Blood sample (*N* = 2237)			Tumor tissue (*N* = 2273)		
	aOR (95% CI)^1^	AUROC (95% CI)	*R*^2^	aOR (95% CI)^1^	AUROC (95% CI)	*R*^2^
*Current vs. Never*						
Chamberlainet al. [12]	10.15 (7.58, 13.59)	0.92 (0.90, 0.94)	0.50	1.09 (0.95, 1.26)	0.53 (0.50, 0.57)	< 0.01
McCartney et al. [14]	27.38 (17.82, 42.06)	0.96 (0.94, 0.97)	0.70	1.45 (1.26, 1.68)	0.58 (0.55, 0.62)	0.02
Elliott et al. [13]	19.85 (13.55, 29.08)	0.94 (0.93, 0.96)	0.56	1.47 (1.28, 1.69)	0.62 (0.59, 0.65)	0.03
Zhang et al. [15]	14.52 (10.51, 20.07)	0.94 (0.92, 0.96)	0.57	1.11 (1.05, 1.18)	0.58 (0.55, 0.62)	0.02
Bollepalli et al. [11]^2^	382.97 (163.26, 898.34)	0.92 (0.90, 0.94)	0.56	1.70 (1.19, 2.43)	0.56 (0.53, 0.59)	0.01
*Former vs. Never*						
Chamberlainet al. [12]	2.28 (1.94, 2.67)	0.66 (0.63, 0.68)	0.06	1.08 (0.98, 1.20)	0.53 (0.51, 0.56)	< 0.01
McCartney et al. [14]	8.50 (6.64, 10.88)	0.80 (0.78, 0.81)	0.22	1.23 (1.11, 1.36)	0.55 (0.52, 0.57)	0.01
Elliott et al. [13]	3.79 (3.14, 4.56)	0.73 (0.71, 0.76)	0.13	1.27 (1.14, 1.41)	0.56 (0.54, 0.59)	0.01
Zhang et al. [15]	3.1 (2.62, 3.66)	0.74 (0.71, 0.76)	0.13	1.07 (1.03, 1.12)	0.54 (0.52, 0.57)	0.01
Bollepalli et al. [11]^2^	2.12 (1.66, 2.72)	0.70 (0.68, 0.72)	0.13	1.15 (0.70, 1.89)	0.55 (0.53, 0.57)	0.01
*Current vs. Former*						
Chamberlainet al. [12]	4.75 (3.86, 5.84)	0.85 (0.82, 0.87)	0.32	0.97 (0.84, 1.12)	0.50 (0.47, 0.54)	< 0.01
McCartney et al. [14]	6.14 (4.92, 7.67)	0.88 (0.85, 0.90)	0.38	1.08 (0.94, 1.24)	0.54 (0.50, 0.57)	< 0.01
Elliott et al. [13]	4.86 (3.96, 5.95)	0.85 (0.82, 0.87)	0.30	1.13 (0.98, 1.30)	0.56 (0.53, 0.60)	< 0.01
Zhang et al. [15]	4.99 (4.05, 6.15)	0.83 (0.81, 0.86)	0.28	1.03 (0.97, 1.09)	0.54 (0.50, 0.57)	< 0.01
Bollepalli et al. [11]^2^	7.16 (4.89, 10.48)	0.75 (0.73, 0.77)	0.18	1.00 (0.52, 1.92)	0.51 (0.49, 0.54)	< 0.01

*OR* Odds ratio, *CI* Confidence interval, *AUROC* Area under the receiver operating characteristic curve. McFadden *R*^2^ measured the explained variation by blood methylation-based smoking panels. *R*^2^ stands for McFadden’s pseudo-*R*^2^. ^1^The multivariable logistic model was adjusted for age, sex, body mass index 5–14 years earlier, alcohol consumption, physical activity, use of nonsteroidal anti-inflammatory drugs, hormone replacement therapy, and prior large bowel endoscopy. ^2^The association between predicted binary smoking status (e.g., predicted current vs. never smokers) and corresponding self-reported binary outcomes was assessed

In contrast, when applying the smoking signatures in tumor methylation data, associations with smoking status were much weaker (Table 2). The point estimates in adjusted logistic models were all below 2, with small McFadden’s pseudo-*R*^2^ (≤ 0.001) and low AUROC values (≤ 0.65). In sensitivity analyses restricted to patients who had not received chemotherapy or radiotherapy, the discriminatory performance of methylation-based smoking signatures for self-reported smoking status remained consistent (Supplement eTable 3).

### Comparison between self-reported and epigenetic smoking signatures in relation to mortality risk

**Fig. 4:**
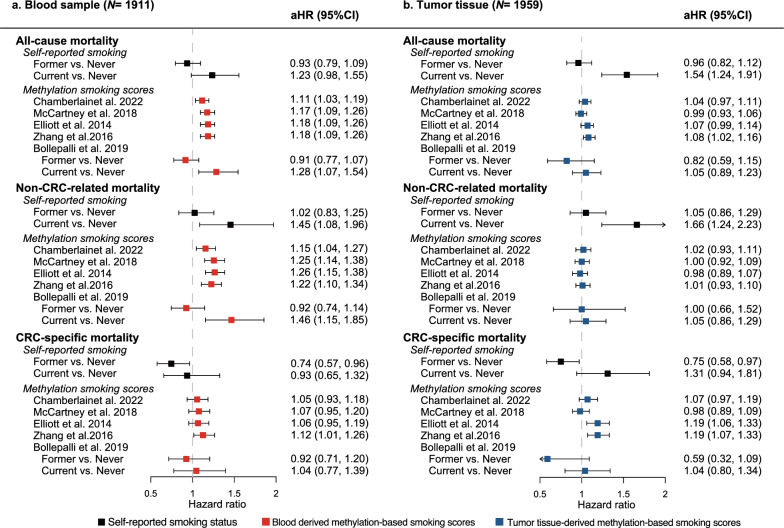
Comparison between self-reported smoking and methylation-based smoking derived from blood and tumor tissue in relation to mortality risk among patients with stage I-III CRC. aHR = adjusted hazards ratio; CI = confidence interval; and CRC = colorectal cancer. The continuous methylation-based scores were standardized. The multivariable Cox regression model was adjusted for age, sex, BMI at diagnosis, physical activity, alcohol consumption, TNM stage, tumor location, and treatment with chemotherapy or radiotherapy

Among 1911 stage I-III patients with blood methylation data (Fig. 4A), self-reported current smokers had a higher all-cause mortality (hazards ratio [HR] 1.23, 95% confidence interval [CI] 0.98–1.55) and non-CRC-related mortality (1.45, 1.08–1.96) compared with never smokers in the fully adjusted models. However, self-reported current smoking was not a risk factor for CRC-specific mortality. Interestingly, self-reported former smokers had a lower CRC-specific mortality compared with never smokers (0.74, 0.57–0.96).

All five methylation-based smoking signatures derived from blood were associated with higher all-cause mortality and non-CRC-related mortality in fully adjusted models. The increased risk of current smokers compared with never smokers, as predicted by the methylation classifier by Bollepalli et al. [11], showed the strongest magnitude of association (all-cause mortality: 1.28, 1.07–1.54; non-CRC-related mortality 1.46, 1.15–1.85). However, significant risk increases were not observed for CRC-specific survival, except for the score developed by Zhang et al. [15] (1.12, 1.01–1.26).

In the 1959 stage I-III CRC patients with tumor methylation data (Fig. 4B), associations of self-reported smoking and mortality were similar to the group with blood samples. However, the methylation signatures were not associated with all-cause or non-CRC-related mortality when applied to tumor tissue. However, two methylation scores, developed by Elliott et al. [13] and Zhang et al. [15], respectively, were significantly associated with CRC-specific mortality (1.19, 1.06–1.33 and 1.19, 1.07–1.33, respectively).

The observed associations were similar when the four continuous methylation scores were dichotomized (Supplement eTable 4). In stage IV CRC patients (Supplement eTable 5), most methylation-based smoking scores were not associated with increased mortality risk, except for the score by Zhang et al. [15], which was strongly associated with non-CRC-related mortality when derived from tumor tissue (2.46, 1.27–4.75).

Significant interactions were observed between four blood-derived methylation scores [12–15] and sex for both all-cause mortality and non-CRC-related mortality (Supplement eTable 6). Additionally, all but the McCartney score [14], showed interactions with age in analyses of non-CRC-related mortality. In tumor tissue samples, only the score by Elliott et al. [13] showed significant interactions with both sex and age for all-cause and non-CRC-related mortality. Stratified analyses revealed higher mortality risk in male and younger patients (Supplement eTable 7). Sensitivity analyses in 2008 overlapping patients with both blood and tissue methylation data showed consistent results with the main analysis (Supplement eTable 8 and eTable 9).

### Methylation-based smoking signatures derived from adjacent normal tissue

None of the five methylation-based smoking signatures showed clear associations with self-reported smoking status when applied to 264 adjacent normal tissue samples (Supplement eTable 10). The signatures were also not associated with mortality risk among stage I-III CRC patients in adjacent normal tissue (eTable 11).

## Discussion

In this large case–control and patient cohort study, we assessed five validated blood methylation-based smoking signatures in predicting smoking status and mortality among CRC patients using blood and tissue samples. Methylation smoking signatures in blood strongly correlated with self-reported smoking, while associations in tumor tissue were weaker. All signatures derived from blood were associated with higher all-cause and non-CRC-related mortality in non-metastatic CRC patients, with a 4-CpG score [15] also associated with CRC-specific mortality. In tumor tissue samples, this score [15] and another 187-CpG score [13] were associated with higher CRC-specific mortality.

All five blood-derived methylation signatures demonstrated strong associations with self-reported smoking status and showed excellent discriminative ability, particularly in differentiating current smokers from never smokers. The limited overlap between CpGs across the evaluated signatures may stem from differences in study populations, measurement platforms, and statistical methods used during their development. The methylation score developed by McCartney et al. [14], which contained the highest number of CpGs (234, of which 224 are unique), performed the best. Similarly, the signatures from Zhang et al. [15], despite comprising only four CpGs, displayed almost perfect discrimination. This suggests that more parsimonious signatures may offer robust performance while reducing redundancy. These results were consistent with findings from the original studies that developed these scores [11–15], as well as other external cohorts [11, 22, 23]. While tumor tissue-derived signatures had weaker associations, they were still mostly statistically significant, highlighting that blood-derived methylation signatures of smoking can even be verified in tumor tissue.

We have, for the first time, demonstrated that blood-derived methylation-based smoking signatures are associated with a higher risk of all-cause mortality among patients with stage I-III CRC. These associations were independent of factors such as age, sex, TNM stage, and other important cofounders. This finding is in line with previous studies on the impact of self-reported smoking on overall survival [3, 5–7]. Our cause-specific analyses showed that the increased risk observed for all-cause mortality was largely due to mortality unrelated to CRC. Indeed, CRC patients who smoke are more likely to develop and die from comorbidities such as cardiovascular and lung diseases [24, 25].

The evidence on the impact of smoking on CRC-specific mortality is mixed. Some studies have found a statistically significantly increased risk of CRC-specific death among current smokers [6, 7], while others have not [26–28]. In our study, only the blood- or tumor-derived 4-CpG smoking signature from Zhang et al. [15] and the tumor-derived 187-CpG smoking score from Elliott et al. [13] were significantly associated with increased CRC-specific mortality. All four CpGs in the Zhang et al. [15] score were also included in the Elliott et al. [13] score. Notably, the cg05575921 CpG site in the *AHRR* gene, a tumor suppressor gene, was consistently associated with smoking and an increased risk of cancer mortality [29–31]. Previous research has shown that the expression of the *AHRR* gene in human colorectal cancer tissue correlates with CD40/CD40L signaling and histological grade [32]. Interestingly, among stage IV patient, the 4-CpG smoking score was also strongly associated with increased risk of non-CRC-related mortality. This further demonstrates the prognostic relevance of this score.

Counterintuitively, our study found that former smokers tend to have lower CRC-specific mortality compared with never smokers. Similarly, a US study reported a protective, though non-significant, association between self-reported former smoking and CRC-specific mortality (HR 0.89, 95%CI 0.72–1.10) [6]. Previous research consistently showed that quitting smoking confers mortality benefits [33–35]. Several factors may explain this finding. Former smokers may develop heightened health consciousness, leading to healthier behaviors, earlier detection, and more diligent medical attention, all of which can improve outcomes [36]. Additionally, if smoking cessation is decades ago, some of the harmful effects and epigenetic changes caused by smoking may reverse or stabilize [7, 37, 38].

Blood-derived methylation signatures outperformed tumor tissue-derived signatures in predicting smoking status, overall mortality, and non-CRC-related mortality. This was expected, as all five signatures were originally developed using blood samples [11–15]. One key explanation lies in the well-known tissue-specific methylation variability of DNA methylation [39]. Blood-derived methylation signatures likely reflect the systemic and cumulative biological effects of smoking, such as chronic inflammation and immune response, which may occur early and persist throughout life [40, 41]. In contrast, methylation changes in tumor tissue may be more variable and dynamic and context-dependent. They can be influenced by factors such as tumor microenvironment (e.g., the presence of stromal cells, immune infiltration), treatment, and disease progression [42, 43]. These differences in cellular composition between tumor tissue and peripheral blood may act as a confounding factor, potentially diluting or obscuring the direct effects of smoking-related epigenetic signals captured in blood. This highlights the importance of accounting for tissue heterogeneity when interpreting the transportability and prognostic utility of methylation-based signatures across different biospecimen types.

Although different methylation platforms were used (EPIC 850k for blood samples and 450k for tumor samples), methodological differences are unlikely to explain the superior performance of blood-based signatures. Because both arrays are based on the same core technology and were preprocessed and normalized using the same pipelines. Only differences in CpG coverage could theoretically influence signature performance. However, all relevant CpG sites were available in the tumor tissue samples, whereas some signatures had partial CpG coverage in blood. Despite this, the blood-derived signatures demonstrated stronger and more consistent associations with smoking behaviors and mortality risks, suggesting that incomplete CpG coverage did not compromise the utility of the blood-based methylation scores.

Nonetheless, two tumor tissue-derived signatures from Elliott et al. [13] and Zhang et al. [15] showed stronger associations with CRC-specific mortality compared to blood-derived signatures. This may be explained by specific components of these scores, such as the cg05575921 site in the *AHRR* gene, that not only reflect smoking exposure but also capture epigenetic changes directly involved in tumor biology, including aspects of the tumor microenvironment, aggressiveness, metastasis potential, and treatment response [32, 44]. However, external validation in independent cohorts with larger sample size and more diverse clinical characteristics is needed to confirm its robustness and clinical utility. If validated, they may offer promising utility as biomarkers for integrating exposure history with tumor behavior in CRC prognostic assessment.

We found that methylation-based smoking signatures derived from adjacent normal tissue were neither associated with self-reported smoking nor with mortality risk in CRC patients. This might be due to the small sample size (only 264 samples) and resulting limited statistical power in this study. Alternatively, adjacent normal tissue may retain the epigenetic patterns similar to healthy tissue and might not show the extensive methylation changes typically associated with smoking, which are more likely to be found in blood (indicative of systemic exposure) or in tumor tissue, where smoking could have contributed to tumorigenesis [44].

We observed significant interactions between blood-derived methylation signatures and sex for both overall mortality and non-CRC-related mortality. It could be partly explained by the higher prevalence of ever smokers among males (68%) compared to females (32%). Additionally, sex-specific hormonal environments, immune responses, and genetic expression profiles might alter the impact of methylation changes on disease progression and mortality [45, 46]. For instance, sex hormones such as estrogen are known to have protective effects against inflammation and oxidative stress, potentially mitigating the adverse effects of smoking-induced aberrant methylation patterns [47]. The stronger associations observed in younger patients might be attributable to age-related epigenetic drift, which may obscure the relationship between specific methylation changes and cancer outcomes in older individuals [48].

To our knowledge, this is the first study to systematically evaluate the prognostic relevance and tissue transportability of established blood-derived smoking-related DNA methylation signatures in CRC. Our approach allowed for a direct comparative evaluation of the same methylation-based smoking scores across blood, tumor, and normal tissues in a large population-based patient cohort with long follow-up time. However, this study has some limitations. First, different methylation platforms were used for blood (Illumina MethylationEPIC 850 k BeadChip) and tissue (Illumina HumanMethylation450 BeadChip) samples. This decision was made based on the availability of the most up-to-date array technology at the time of measurement. While this might introduce concerns regarding the technical variability and comparability, both arrays are based on the same core technology, including bisulfite conversion, probe chemistry, and scanning methods [49]. Furthermore, we applied the same preprocessing and normalization pipelines across both platforms to ensure methodological uniformity.

Second, although we adjusted for several potential confounders, residual confounding by unmeasured variables (e.g., comorbid of lung disease, detailed treatment regimens) could still influence the outcomes. Third, the sample size for adjacent normal tissue was significantly smaller compared to blood and tumor tissue samples, which may limit the statistical power to detect true associations in adjacent normal tissue. Fourth, the patient samples for the blood-based analyses and the tissue-based analyses were not the same, even though they largely overlapped and were almost identical in size. Lastly, the DACHS cohort is limited to German-speaking participants from a specific geographic region in Germany, which may limit the generalizability of our findings.

Further research is needed to explore the utility of these methylation-based smoking signatures across diverse populations and various cancer types to establish their generalizability and applicability in different clinical settings. Investigating the biological mechanisms underpinning the differences in methylation signatures between blood and tumor tissue could provide insights into the distinct roles of systemic versus localized epigenetic changes in CRC progression and mortality. Integrating promising methylation-based smoking signatures (e.g., the 4-CpG score from Zhang et al. [15]) with other biomarkers and clinical variables may enhance predictive models for CRC prognosis and contribute to the development of comprehensive, multi-modal predictive tools.

## Conclusions

In conclusion, our results highlight the utility of blood-derived methylation signatures as effective biomarkers for refining the quantification of smoking exposure and assessing its impact on mortality risk in non-metastatic CRC patients. While the transferability of these signatures to tissue samples is limited, several signatures [11, 13, 15] still demonstrate relevance to CRC-specific mortality. These findings underscore the potential of methylation-based smoking markers as noninvasive, objective tools for assessing smoking exposure and its impact on cancer prognosis.

## Supplementary Information

Additional file1 (DOCX 317 KB)

## Data Availability

The datasets used and analyzed during the current study are available from the corresponding author on reasonable request.

## Abbreviations

CRC: Colorectal cancer
STROBE: The Strengthening the Reporting of Observational Studies in Epidemiology Statement
TNM: Tumor, lymph node, metastasis staging system
AUROC: The area under the receiver operating characteristic curve
BMI: Body mass index
IQR: Interquartile range
