# Supplementary material for: Methylation-based smoking signatures in blood and tissue samples for the prediction of self-reported smoking status and mortality in patients with colorectal cancer
Source: Clin Epigenetics. 2025 Jul 3;17:113. doi: 10.1186/s13148-025-01918-9 (PMC12225191; doi:10.1186/s13148-025-01918-9)
Supplement: Supplementary file 1 — Additional file1 (DOCX 317 KB) [file 13148_2025_1918_MOESM1_ESM.docx]

[eTable 1. The percentage of available CpG sites included in smoking signatures on both arrays 1](#_Toc198295719)

[eTable 2. Distribution of five methylation-based scores and individual CpGs by sample sources 2](#_Toc198295720)

[eFigure1 Distribution of self-reported smoking status and methylation-based smoking signatures/status by TNM stage 16](#_Toc198295721)

[eTable 3. Association between methylation-based smoking scores and self-reported smoking status among patients without treatment with chemotherapy or radiotherapy 17](#_Toc198295722)

[eTable 4. Sensitivity analyses for the associations between dichotomized methylation-based scores and mortality among patients with stage I-III CRC 18](#_Toc198295723)

[eTable 5. The associations between methylation-based smoking signatures and mortality among patients with stage IV CRC 19](#_Toc198295724)

[eTable 6. Interaction effects of methylation-based smoking signatures with sex and age on mortality outcomes 20](#_Toc198295725)

[eTable 7. Stratified analyses for methylation-based signatures showing significant interaction effect with age or sex 21](#_Toc198295726)

[eTable 8. Associations between methylation-based smoking scores and self-reported smoking status among overlapping patients with both blood and tumor sample 22](#_Toc198295727)

[eTable 9. Associations between methylation-based smoking scores and CRC mortality among overlapping stage I-III patients with both blood and tumor methylation data 23](#_Toc198295728)

[eTable 10. Associations between methylation-based smoking scores derived from adjacent normal tissue and self-reported smoking status 24](#_Toc198295729)

[eTable 11. Associations between methylation-based smoking scores derived from adjacent normal tissue and mortality 25](#_Toc198295730)

##

## eTable 1. The percentage of available CpG sites included in smoking signatures on both arrays

|  |  |  | **Available CpGs (%)** | |
| --- | --- | --- | --- | --- |
| **Methylation-based smoking signatures** | **Normalization method** | **No.CpGs** | **Blood^1^** | **Tissue^2^** |
| McCartney et al. 2018 | QN | 233 | 233 (100%) | 233 (100%) |
| Chamberlain et al. 2002 | QN | 5 | 5 (100%) | 5 (100%) |
| Elliott et al. 2014 | SQN | 187 | 174 (93%) | 187 (100%) |
| Zhang et al. 2016 | ILM | 4 | 2 (50%) | 4 (100%) |
| Bollepalli et al. 2019 | QN | 121 | 111(91%) | 121 (100%) |

QN = Quantile Normalization, SQN = Subset Quantile Normalization, ILM = Illumina Normalization.

^1^MethylationEPIC 850k BeadChip was used for for peripheral blood sample

^2^ Illumina HumanMethylation450 BeadChip was used for tumor tissue and

## eTable 2. Distribution of five methylation-based scores and individual CpGs by sample sources

| **Score/CpG sites** | **Blood sample  (N = 2237)** | **Tumor sample  (N = 2273)** | ***P* value** |
| --- | --- | --- | --- |
| **McCartney et al. 2018** | **2.345 (2.091, 2.832)** | **2.626 (2.133, 3.115)** | <0.001 |
| cg10573386 | 0.175 (0.154, 0.199) | 0.102 (0.084, 0.183) | <0.001 |
| cg13560072 | 1.197 (1.189, 1.204) | 1.110 (1.063, 1.147) | <0.001 |
| cg11084015 | 1.128 (1.119, 1.138) | 1.075 (1.050, 1.097) | <0.001 |
| cg10321266 | 0.064 (0.060, 0.069) | 0.063 (0.055, 0.074) | 0.002 |
| cg07597069 | 0.796 (0.792, 0.800) | 0.817 (0.806, 0.826) | <0.001 |
| cg05218653 | 0.789 (0.784, 0.794) | 0.612 (0.449, 0.696) | <0.001 |
| cg00077898 | 0.786 (0.780, 0.791) | 0.761 (0.746, 0.771) | <0.001 |
| cg11207515 | 0.286 (0.245, 0.326) | 0.180 (0.149, 0.221) | <0.001 |
| cg18392085 | 0.053 (0.045, 0.062) | 0.039 (0.034, 0.046) | <0.001 |
| cg06088918 | 0.106 (0.090, 0.124) | 0.052 (0.044, 0.063) | <0.001 |
| cg20077343 | 0.589 (0.582, 0.594) | 0.394 (0.334, 0.453) | <0.001 |
| cg24169820 | 0.585 (0.583, 0.588) | 0.529 (0.494, 0.557) | <0.001 |
| cg17833862 | 0.533 (0.530, 0.536) | 0.496 (0.469, 0.514) | <0.001 |
| cg23079012 | 0.504 (0.492, 0.513) | 0.443 (0.396, 0.469) | <0.001 |
| cg10696199 | 0.034 (0.031, 0.038) | 0.044 (0.039, 0.050) | <0.001 |
| cg05442408 | 0.054 (0.049, 0.061) | 0.268 (0.226, 0.305) | <0.001 |
| cg08038054 | 0.166 (0.146, 0.190) | 0.223 (0.183, 0.262) | <0.001 |
| cg26775087 | 0.337 (0.329, 0.343) | 0.221 (0.196, 0.245) | <0.001 |
| cg22371743 | 0.028 (0.025, 0.031) | 0.025 (0.022, 0.030) | <0.001 |
| cg04136748 | 0.216 (0.207, 0.223) | 0.188 (0.174, 0.202) | <0.001 |
| cg23288337 | 0.109 (0.102, 0.116) | 0.056 (0.048, 0.067) | <0.001 |
| cg04561727 | 0.242 (0.234, 0.251) | 0.165 (0.128, 0.196) | <0.001 |
| cg09648091 | 0.020 (0.016, 0.024) | 0.024 (0.020, 0.029) | <0.001 |
| cg14142965 | 0.023 (0.019, 0.029) | 0.034 (0.023, 0.064) | <0.001 |
| cg14708990 | 0.214 (0.202, 0.226) | 0.102 (0.085, 0.127) | <0.001 |
| cg01558110 | 0.245 (0.239, 0.249) | 0.160 (0.137, 0.184) | <0.001 |
| cg11704876 | 0.181 (0.172, 0.192) | 0.212 (0.190, 0.226) | <0.001 |
| cg03616722 | 0.273 (0.265, 0.278) | 0.153 (0.102, 0.196) | <0.001 |
| cg24714011 | 0.035 (0.029, 0.040) | 0.027 (0.024, 0.031) | <0.001 |
| cg00231810 | 0.245 (0.240, 0.249) | 0.196 (0.177, 0.212) | <0.001 |
| cg10506497 | 0.224 (0.222, 0.225) | 0.229 (0.215, 0.236) | <0.001 |
| cg22872778 | 0.232 (0.229, 0.235) | 0.210 (0.199, 0.218) | <0.001 |
| cg10752315 | 0.199 (0.194, 0.203) | 0.097 (0.082, 0.117) | <0.001 |
| cg05609159 | 0.108 (0.100, 0.117) | 0.064 (0.052, 0.080) | <0.001 |
| cg16595261 | 0.015 (0.012, 0.018) | 0.019 (0.015, 0.024) | <0.001 |
| cg05852477 | 0.193 (0.192, 0.195) | 0.145 (0.123, 0.162) | <0.001 |
| cg00585474 | 0.016 (0.014, 0.018) | 0.023 (0.020, 0.027) | <0.001 |
| cg18881501 | 0.050 (0.042, 0.059) | 0.036 (0.029, 0.045) | <0.001 |
| cg04834228 | 0.154 (0.152, 0.156) | 0.096 (0.084, 0.105) | <0.001 |
| cg13914990 | 0.191 (0.189, 0.194) | 0.180 (0.175, 0.184) | <0.001 |
| cg12205822 | 0.194 (0.193, 0.196) | 0.175 (0.169, 0.180) | <0.001 |
| cg09462089 | 0.157 (0.154, 0.160) | 0.145 (0.138, 0.151) | <0.001 |
| cg26394282 | 0.146 (0.142, 0.149) | 0.109 (0.096, 0.119) | <0.001 |
| cg17479718 | 0.156 (0.155, 0.157) | 0.110 (0.100, 0.119) | <0.001 |
| cg27222803 | 0.009 (0.007, 0.010) | 0.006 (0.005, 0.007) | <0.001 |
| cg16395614 | 0.148 (0.145, 0.151) | 0.147 (0.144, 0.149) | <0.001 |
| cg08189186 | 0.076 (0.070, 0.081) | 0.097 (0.081, 0.113) | <0.001 |
| cg20037507 | 0.093 (0.090, 0.097) | 0.103 (0.091, 0.112) | <0.001 |
| cg20543544 | 0.054 (0.045, 0.063) | 0.097 (0.090, 0.102) | <0.001 |
| cg19770748 | 0.135 (0.134, 0.135) | 0.083 (0.067, 0.098) | <0.001 |
| cg17201651 | 0.097 (0.092, 0.100) | 0.041 (0.034, 0.050) | <0.001 |
| cg08376310 | 0.120 (0.117, 0.123) | 0.079 (0.059, 0.096) | <0.001 |
| cg01518225 | 0.067 (0.062, 0.073) | 0.098 (0.089, 0.104) | <0.001 |
| cg03984405 | 0.112 (0.109, 0.114) | 0.088 (0.080, 0.094) | <0.001 |
| cg12486537 | 0.010 (0.008, 0.012) | 0.060 (0.026, 0.074) | <0.001 |
| cg20811236 | 0.067 (0.062, 0.073) | 0.063 (0.054, 0.074) | <0.001 |
| cg18050634 | 0.097 (0.095, 0.099) | 0.092 (0.074, 0.100) | <0.001 |
| cg15235798 | 0.052 (0.050, 0.055) | 0.056 (0.031, 0.073) | <0.001 |
| cg27305917 | 0.047 (0.042, 0.053) | 0.036 (0.030, 0.043) | <0.001 |
| cg12723292 | 0.004 (0.003, 0.005) | 0.006 (0.006, 0.007) | <0.001 |
| cg19033444 | 0.084 (0.082, 0.086) | 0.049 (0.042, 0.056) | <0.001 |
| cg01933695 | 0.045 (0.042, 0.049) | 0.020 (0.017, 0.025) | <0.001 |
| cg03423767 | 0.061 (0.057, 0.066) | 0.061 (0.051, 0.070) | 0.309 |
| cg09341154 | 0.046 (0.042, 0.049) | 0.043 (0.029, 0.054) | <0.001 |
| cg05168580 | 0.020 (0.017, 0.023) | 0.025 (0.019, 0.031) | <0.001 |
| cg07894334 | 0.074 (0.071, 0.077) | 0.016 (0.013, 0.020) | <0.001 |
| cg19920183 | 0.006 (0.005, 0.007) | 0.007 (0.006, 0.008) | <0.001 |
| cg12945194 | 0.081 (0.080, 0.082) | 0.058 (0.052, 0.062) | <0.001 |
| cg07933378 | 0.075 (0.069, 0.079) | 0.031 (0.028, 0.034) | <0.001 |
| cg00145103 | 0.021 (0.018, 0.024) | 0.016 (0.014, 0.020) | <0.001 |
| cg02229382 | 0.033 (0.030, 0.036) | 0.039 (0.030, 0.050) | <0.001 |
| cg20012848 | 0.067 (0.066, 0.068) | 0.056 (0.053, 0.060) | <0.001 |
| cg15043384 | 0.066 (0.064, 0.068) | 0.055 (0.049, 0.060) | <0.001 |
| cg23279756 | 0.018 (0.017, 0.021) | 0.055 (0.049, 0.059) | <0.001 |
| cg15939523 | 0.065 (0.060, 0.067) | 0.062 (0.058, 0.064) | <0.001 |
| cg09359017 | 0.066 (0.065, 0.067) | 0.047 (0.041, 0.052) | <0.001 |
| cg14250560 | 0.043 (0.040, 0.045) | 0.060 (0.057, 0.064) | <0.001 |
| cg25037335 | 0.055 (0.051, 0.059) | 0.058 (0.052, 0.063) | <0.001 |
| cg04418355 | 0.051 (0.044, 0.055) | 0.030 (0.026, 0.034) | <0.001 |
| cg07520269 | 0.029 (0.023, 0.034) | 0.019 (0.015, 0.028) | <0.001 |
| cg08926642 | 0.060 (0.058, 0.062) | 0.061 (0.057, 0.063) | <0.001 |
| cg14532376 | 0.061 (0.061, 0.062) | 0.054 (0.051, 0.056) | <0.001 |
| cg05720891 | 0.066 (0.065, 0.066) | 0.040 (0.032, 0.047) | <0.001 |
| cg04060128 | 0.008 (0.006, 0.012) | 0.021 (0.016, 0.026) | <0.001 |
| cg21357361 | 0.003 (0.003, 0.004) | 0.005 (0.004, 0.006) | <0.001 |
| cg23294158 | 0.025 (0.024, 0.026) | 0.012 (0.010, 0.014) | <0.001 |
| cg23657179 | 0.030 (0.025, 0.034) | 0.029 (0.022, 0.035) | <0.001 |
| cg07708286 | 0.060 (0.059, 0.060) | 0.049 (0.045, 0.052) | <0.001 |
| cg21476494 | 0.022 (0.018, 0.026) | 0.039 (0.033, 0.044) | <0.001 |
| cg05634428 | 0.039 (0.036, 0.041) | 0.040 (0.036, 0.044) | <0.001 |
| cg02981731 | 0.018 (0.015, 0.020) | 0.018 (0.011, 0.027) | 0.769 |
| cg05251669 | 0.033 (0.025, 0.038) | 0.024 (0.016, 0.034) | <0.001 |
| cg26644049 | 0.029 (0.027, 0.030) | 0.031 (0.029, 0.034) | <0.001 |
| cg06530441 | 0.026 (0.024, 0.027) | 0.021 (0.017, 0.025) | <0.001 |
| cg26047066 | 0.050 (0.049, 0.050) | 0.047 (0.044, 0.048) | <0.001 |
| cg19850024 | 0.045 (0.044, 0.047) | 0.044 (0.040, 0.047) | <0.001 |
| cg22709563 | 0.008 (0.006, 0.009) | 0.012 (0.010, 0.018) | <0.001 |
| cg00449767 | 0.042 (0.041, 0.043) | 0.037 (0.033, 0.040) | <0.001 |
| cg00340052 | 0.039 (0.037, 0.041) | 0.042 (0.040, 0.044) | <0.001 |
| cg05857996 | 0.037 (0.035, 0.039) | 0.042 (0.040, 0.044) | <0.001 |
| cg17221856 | 0.039 (0.038, 0.040) | 0.030 (0.025, 0.032) | <0.001 |
| cg08363339 | 0.036 (0.034, 0.038) | 0.016 (0.013, 0.019) | <0.001 |
| cg19863595 | 0.036 (0.035, 0.036) | 0.028 (0.025, 0.032) | <0.001 |
| cg05265884 | 0.021 (0.019, 0.023) | 0.028 (0.025, 0.030) | <0.001 |
| cg10090414 | 0.036 (0.035, 0.037) | 0.027 (0.025, 0.029) | <0.001 |
| cg04191989 | 0.035 (0.034, 0.035) | 0.031 (0.029, 0.033) | <0.001 |
| cg08896053 | 0.005 (0.004, 0.005) | 0.015 (0.009, 0.021) | <0.001 |
| cg14752227 | 0.035 (0.029, 0.036) | 0.028 (0.022, 0.033) | <0.001 |
| cg16204818 | 0.005 (0.004, 0.006) | 0.025 (0.022, 0.028) | <0.001 |
| cg09776718 | 0.033 (0.032, 0.033) | 0.030 (0.029, 0.032) | <0.001 |
| cg10143416 | 0.031 (0.031, 0.031) | 0.026 (0.024, 0.028) | <0.001 |
| cg14294708 | 0.005 (0.004, 0.007) | 0.003 (0.002, 0.004) | <0.001 |
| cg13953753 | 0.026 (0.025, 0.027) | 0.028 (0.027, 0.028) | <0.001 |
| cg18336825 | 0.003 (0.003, 0.004) | 0.003 (0.002, 0.003) | <0.001 |
| cg18153977 | 0.007 (0.005, 0.011) | 0.002 (0.002, 0.002) | <0.001 |
| cg18568930 | 0.020 (0.018, 0.021) | 0.013 (0.010, 0.017) | <0.001 |
| cg08570243 | 0.005 (0.003, 0.008) | 0.003 (0.002, 0.005) | <0.001 |
| cg00950381 | 0.017 (0.017, 0.018) | 0.019 (0.018, 0.020) | <0.001 |
| cg18758559 | 0.017 (0.016, 0.018) | 0.009 (0.008, 0.012) | <0.001 |
| cg24870966 | 0.015 (0.014, 0.016) | 0.005 (0.004, 0.006) | <0.001 |
| cg08466269 | 0.015 (0.015, 0.015) | 0.014 (0.014, 0.015) | <0.001 |
| cg14071650 | 0.007 (0.007, 0.008) | 0.008 (0.006, 0.010) | <0.001 |
| cg03464336 | 0.004 (0.003, 0.005) | 0.003 (0.003, 0.004) | <0.001 |
| cg09154837 | 0.001 (0.001, 0.001) | 0.001 (0.001, 0.001) | <0.001 |
| cg23832388 | 0.009 (0.008, 0.010) | 0.006 (0.005, 0.007) | <0.001 |
| cg25740457 | 0.007 (0.006, 0.008) | 0.010 (0.009, 0.012) | <0.001 |
| cg05543456 | 0.012 (0.012, 0.013) | 0.011 (0.011, 0.012) | <0.001 |
| cg07318204 | 0.012 (0.012, 0.012) | 0.010 (0.009, 0.011) | <0.001 |
| cg01074657 | 0.005 (0.004, 0.006) | 0.002 (0.002, 0.003) | <0.001 |
| cg00853687 | 0.013 (0.012, 0.013) | 0.010 (0.008, 0.011) | <0.001 |
| cg18712599 | 0.012 (0.012, 0.012) | 0.009 (0.008, 0.010) | <0.001 |
| cg01175394 | 0.012 (0.012, 0.012) | 0.008 (0.007, 0.009) | <0.001 |
| cg08399733 | 0.009 (0.009, 0.009) | 0.006 (0.005, 0.006) | <0.001 |
| cg24261754 | 0.000 (0.000, 0.001) | 0.001 (0.001, 0.002) | <0.001 |
| cg14778711 | 0.001 (0.001, 0.001) | 0.001 (0.001, 0.001) | <0.001 |
| cg05046722 | 0.006 (0.006, 0.007) | 0.004 (0.003, 0.005) | <0.001 |
| cg20235075 | 0.004 (0.004, 0.004) | 0.004 (0.003, 0.004) | 0.556 |
| cg26744079 | 0.005 (0.004, 0.005) | 0.004 (0.004, 0.005) | <0.001 |
| cg16900209 | 0.001 (0.000, 0.001) | 0.002 (0.002, 0.003) | <0.001 |
| cg17702370 | 0.001 (0.001, 0.001) | 0.001 (0.001, 0.001) | <0.001 |
| cg12097672 | 0.005 (0.005, 0.005) | 0.004 (0.004, 0.004) | <0.001 |
| cg26996890 | 0.004 (0.004, 0.004) | 0.004 (0.004, 0.004) | <0.001 |
| cg12253071 | 0.003 (0.003, 0.003) | 0.002 (0.002, 0.002) | <0.001 |
| cg15209053 | 0.003 (0.003, 0.003) | 0.002 (0.002, 0.003) | <0.001 |
| cg10861731 | 0.002 (0.001, 0.002) | 0.001 (0.001, 0.002) | <0.001 |
| cg00011924 | 0.001 (0.001, 0.001) | 0.001 (0.001, 0.001) | <0.001 |
| cg10623600 | 0.001 (0.000, 0.001) | 0.001 (0.001, 0.001) | <0.001 |
| cg03256090 | 0.000 (0.000, 0.000) | 0.000 (0.000, 0.000) | <0.001 |
| cg24519533 | 0.001 (0.001, 0.001) | 0.001 (0.001, 0.001) | <0.001 |
| cg15094236 | 0.000 (0.000, 0.000) | 0.000 (0.000, 0.000) | <0.001 |
| cg14523898 | 0.000 (0.000, 0.000) | 0.000 (0.000, 0.000) | <0.001 |
| cg08617581 | -0.001 (-0.001, -0.001) | -0.001 (-0.001, -0.001) | <0.001 |
| cg09977703 | -0.001 (-0.001, -0.001) | -0.001 (-0.002, -0.001) | <0.001 |
| cg23332900 | -0.001 (-0.001, -0.001) | -0.001 (-0.002, -0.001) | <0.001 |
| cg05783384 | -0.003 (-0.004, -0.003) | -0.002 (-0.002, -0.002) | <0.001 |
| cg05030953 | -0.002 (-0.003, -0.001) | -0.003 (-0.004, -0.003) | <0.001 |
| cg03354772 | -0.001 (-0.001, -0.001) | -0.003 (-0.003, -0.002) | <0.001 |
| cg08079266 | -0.010 (-0.010, -0.010) | -0.007 (-0.008, -0.007) | <0.001 |
| cg14788994 | -0.011 (-0.011, -0.011) | -0.009 (-0.010, -0.009) | <0.001 |
| cg14071524 | -0.010 (-0.010, -0.009) | -0.007 (-0.008, -0.005) | <0.001 |
| cg18155226 | -0.001 (-0.001, -0.001) | -0.001 (-0.001, -0.001) | <0.001 |
| cg13937905 | -0.012 (-0.013, -0.012) | -0.006 (-0.007, -0.005) | <0.001 |
| cg04520169 | -0.007 (-0.008, -0.005) | -0.009 (-0.011, -0.003) | <0.001 |
| cg01139016 | -0.012 (-0.012, -0.011) | -0.008 (-0.009, -0.007) | <0.001 |
| cg06840659 | -0.003 (-0.003, -0.002) | -0.002 (-0.002, -0.001) | <0.001 |
| cg18126613 | -0.013 (-0.014, -0.011) | -0.008 (-0.011, -0.007) | <0.001 |
| cg09592244 | -0.005 (-0.007, -0.003) | -0.006 (-0.010, -0.004) | <0.001 |
| cg02960500 | -0.026 (-0.027, -0.024) | -0.019 (-0.021, -0.017) | <0.001 |
| cg00929860 | -0.032 (-0.033, -0.031) | -0.032 (-0.033, -0.031) | <0.001 |
| cg02539153 | -0.025 (-0.026, -0.023) | -0.026 (-0.028, -0.023) | <0.001 |
| cg19508967 | -0.034 (-0.034, -0.033) | -0.016 (-0.018, -0.014) | <0.001 |
| cg07266910 | -0.023 (-0.025, -0.021) | -0.027 (-0.028, -0.025) | <0.001 |
| cg19854213 | -0.036 (-0.037, -0.036) | -0.034 (-0.035, -0.033) | <0.001 |
| cg24729221 | -0.023 (-0.026, -0.021) | -0.030 (-0.032, -0.028) | <0.001 |
| cg26282792 | -0.019 (-0.021, -0.018) | -0.031 (-0.034, -0.028) | <0.001 |
| cg19140375 | -0.031 (-0.033, -0.029) | -0.033 (-0.038, -0.026) | <0.001 |
| cg16780015 | -0.046 (-0.048, -0.043) | -0.026 (-0.031, -0.023) | <0.001 |
| cg17372758 | -0.051 (-0.052, -0.050) | -0.022 (-0.028, -0.018) | <0.001 |
| cg08654020 | -0.027 (-0.029, -0.025) | -0.012 (-0.014, -0.010) | <0.001 |
| cg19450714 | -0.017 (-0.019, -0.014) | -0.011 (-0.015, -0.009) | <0.001 |
| cg20764780 | -0.040 (-0.043, -0.036) | -0.023 (-0.028, -0.020) | <0.001 |
| cg20052079 | -0.031 (-0.034, -0.028) | -0.035 (-0.039, -0.030) | <0.001 |
| cg06624358 | -0.029 (-0.031, -0.027) | -0.023 (-0.026, -0.020) | <0.001 |
| cg23560546 | -0.031 (-0.033, -0.028) | -0.024 (-0.028, -0.021) | <0.001 |
| cg08311343 | -0.055 (-0.059, -0.052) | -0.018 (-0.022, -0.014) | <0.001 |
| cg20402783 | -0.033 (-0.037, -0.029) | -0.047 (-0.051, -0.041) | <0.001 |
| cg09007290 | -0.074 (-0.076, -0.071) | -0.073 (-0.075, -0.070) | <0.001 |
| cg13726504 | -0.069 (-0.070, -0.068) | -0.049 (-0.051, -0.046) | <0.001 |
| cg22659262 | -0.056 (-0.060, -0.052) | -0.044 (-0.059, -0.031) | <0.001 |
| cg06644428 | -0.023 (-0.028, -0.016) | -0.048 (-0.058, -0.035) | <0.001 |
| cg19895882 | -0.005 (-0.006, -0.004) | -0.004 (-0.005, -0.003) | <0.001 |
| cg16734433 | -0.085 (-0.089, -0.082) | -0.049 (-0.059, -0.041) | <0.001 |
| cg12411704 | -0.086 (-0.089, -0.083) | -0.077 (-0.083, -0.069) | <0.001 |
| cg08447324 | -0.015 (-0.019, -0.013) | -0.053 (-0.065, -0.031) | <0.001 |
| cg12745203 | -0.019 (-0.021, -0.017) | -0.057 (-0.065, -0.049) | <0.001 |
| cg07340580 | -0.031 (-0.033, -0.029) | -0.014 (-0.028, -0.010) | <0.001 |
| cg05387119 | -0.091 (-0.092, -0.090) | -0.053 (-0.066, -0.042) | <0.001 |
| cg17271056 | -0.078 (-0.083, -0.073) | -0.088 (-0.092, -0.084) | <0.001 |
| cg14649424 | -0.088 (-0.090, -0.086) | -0.088 (-0.093, -0.082) | 0.462 |
| cg07958878 | -0.102 (-0.103, -0.102) | -0.091 (-0.095, -0.086) | <0.001 |
| cg04232649 | -0.007 (-0.009, -0.006) | -0.020 (-0.026, -0.015) | <0.001 |
| cg25755191 | -0.030 (-0.041, -0.022) | -0.026 (-0.035, -0.020) | <0.001 |
| cg17414900 | -0.015 (-0.017, -0.014) | -0.009 (-0.010, -0.008) | <0.001 |
| cg05533953 | -0.010 (-0.011, -0.009) | -0.013 (-0.019, -0.011) | <0.001 |
| cg02563952 | -0.031 (-0.035, -0.027) | -0.022 (-0.026, -0.019) | <0.001 |
| cg01798157 | -0.058 (-0.066, -0.049) | -0.042 (-0.064, -0.033) | <0.001 |
| cg24886257 | -0.075 (-0.093, -0.062) | -0.138 (-0.144, -0.129) | <0.001 |
| cg05057777 | -0.105 (-0.117, -0.090) | -0.090 (-0.104, -0.072) | <0.001 |
| cg10592478 | -0.165 (-0.168, -0.162) | -0.078 (-0.097, -0.062) | <0.001 |
| cg17287155 | -0.167 (-0.171, -0.162) | -0.149 (-0.155, -0.142) | <0.001 |
| cg13808240 | -0.018 (-0.020, -0.016) | -0.028 (-0.031, -0.025) | <0.001 |
| cg18758585 | -0.034 (-0.038, -0.030) | -0.132 (-0.146, -0.119) | <0.001 |
| cg01940273 | -0.140 (-0.149, -0.129) | -0.150 (-0.167, -0.129) | <0.001 |
| cg11560197 | -0.112 (-0.122, -0.102) | -0.151 (-0.164, -0.136) | <0.001 |
| cg21322436 | -0.128 (-0.134, -0.121) | -0.191 (-0.204, -0.171) | <0.001 |
| cg12126786 | -0.017 (-0.019, -0.015) | -0.058 (-0.066, -0.048) | <0.001 |
| cg15551292 | -0.021 (-0.024, -0.017) | -0.022 (-0.026, -0.019) | <0.001 |
| cg25542733 | -0.050 (-0.058, -0.042) | -0.026 (-0.030, -0.023) | <0.001 |
| cg04027043 | -0.381 (-0.388, -0.371) | -0.123 (-0.170, -0.096) | <0.001 |
| cg18754985 | -0.390 (-0.395, -0.384) | -0.342 (-0.356, -0.326) | <0.001 |
| cg06241380 | -0.032 (-0.036, -0.028) | -0.029 (-0.035, -0.024) | <0.001 |
| cg19452873 | -0.034 (-0.039, -0.029) | -0.050 (-0.058, -0.042) | <0.001 |
| cg19859270 | -0.457 (-0.465, -0.449) | -0.401 (-0.422, -0.373) | <0.001 |
| cg24213508 | -0.120 (-0.147, -0.098) | -0.095 (-0.113, -0.076) | <0.001 |
| cg03342084 | -0.019 (-0.024, -0.015) | -0.026 (-0.032, -0.021) | <0.001 |
| cg20257821 | -0.533 (-0.537, -0.529) | -0.399 (-0.421, -0.372) | <0.001 |
| cg20560182 | -0.023 (-0.027, -0.020) | -0.029 (-0.035, -0.025) | <0.001 |
| cg16398761 | -0.113 (-0.128, -0.098) | -0.200 (-0.234, -0.170) | <0.001 |
| cg12651044 | -0.064 (-0.073, -0.055) | -0.069 (-0.082, -0.059) | <0.001 |
| cg03636183 | -0.625 (-0.655, -0.579) | -0.567 (-0.648, -0.494) | <0.001 |
| cg25013095 | -1.239 (-1.250, -1.228) | -1.172 (-1.198, -1.140) | <0.001 |
| cg21566642 | -1.748 (-1.803, -1.669) | -1.514 (-1.617, -1.363) | <0.001 |
| cg05575921 | -4.710 (-4.856, -4.309) | -3.252 (-3.712, -2.754) | <0.001 |
| **Chamberlain et al. 2022** | **18.959 (17.755, 20.322)** | **22.107 (20.512, 23.973)** | <0.001 |
| cg05575921 | -9.861 (-10.166, -9.020) | -6.809 (-7.771, -5.764) | <0.001 |
| cg26703534 | -17.055 (-17.658, -16.421) | -19.020 (-19.814, -17.993) | <0.001 |
| cg23480021 | 2.186 (1.796, 2.581) | 2.530 (2.222, 2.805) | <0.001 |
| cg08118908 | 41.575 (40.947, 42.221) | 41.901 (41.049, 42.694) | <0.001 |
| cg00336149 | 1.976 (1.706, 2.260) | 3.570 (3.281, 3.844) | <0.001 |
| **Elliott et al. 2014** | **-2.724 (-4.543, -0.079)** | **14.048 (11.769, 16.136)** | **<0.001** |
| cg09469355 | 0.425 (0.402, 0.451) | 0.178 (0.147, 0.218) | <0.001 |
| cg08884752 | 0.638 (0.599, 0.676) | 0.231 (0.182, 0.299) | <0.001 |
| cg12547807 | 0.332 (0.300, 0.363) | 0.247 (0.197, 0.306) | <0.001 |
| cg04885881 | 0.389 (0.349, 0.426) | 0.297 (0.243, 0.352) | <0.001 |
| cg21393163 | 0.099 (0.081, 0.119) | 0.285 (0.230, 0.349) | <0.001 |
| cg21913886 | 0.791 (0.759, 0.818) | 0.689 (0.628, 0.732) | <0.001 |
| cg19713429 | 0.211 (0.184, 0.242) | 0.185 (0.132, 0.284) | <0.001 |
| cg27537125 | 0.185 (0.163, 0.209) | 0.183 (0.140, 0.250) | 0.648 |
| cg15542713 | 0.501 (0.443, 0.558) | 0.532 (0.451, 0.601) | <0.001 |
| cg24049493 | 0.251 (0.197, 0.309) | 0.433 (0.310, 0.541) | <0.001 |
| cg23090529 | 0.333 (0.285, 0.380) | 0.623 (0.557, 0.684) | <0.001 |
| cg21140898 | 0.502 (0.457, 0.541) | 0.700 (0.642, 0.748) | <0.001 |
| cg19406367 | 0.689 (0.651, 0.726) | 0.662 (0.589, 0.718) | <0.001 |
| cg25189904 | 0.381 (0.328, 0.427) | 0.189 (0.150, 0.236) | <0.001 |
| cg09662411 | 0.742 (0.715, 0.766) | 0.528 (0.445, 0.589) | <0.001 |
| cg18146737 | 0.881 (0.856, 0.892) | 0.679 (0.581, 0.734) | <0.001 |
| cg12876356 | 0.757 (0.720, 0.779) | 0.473 (0.340, 0.602) | <0.001 |
| cg18316974 | 0.861 (0.849, 0.871) | 0.765 (0.654, 0.821) | <0.001 |
| cg09935388 | 0.686 (0.627, 0.727) | 0.508 (0.380, 0.632) | <0.001 |
| cg11231349 | NA (NA, NA) | 0.426 (0.362, 0.494) | NA |
| cg08709672 | 0.606 (0.572, 0.641) | 0.453 (0.392, 0.511) | <0.001 |
| cg20295214 | 0.742 (0.719, 0.763) | 0.732 (0.686, 0.772) | <0.001 |
| cg03547355 | 0.530 (0.508, 0.549) | 0.191 (0.156, 0.232) | <0.001 |
| cg17819085 | 0.660 (0.636, 0.681) | 0.253 (0.215, 0.305) | <0.001 |
| cg23079012 | 0.901 (0.879, 0.917) | 0.791 (0.709, 0.838) | <0.001 |
| cg06635952 | 0.291 (0.254, 0.339) | 0.232 (0.187, 0.318) | <0.001 |
| cg26271591 | 0.304 (0.270, 0.345) | 0.262 (0.203, 0.332) | <0.001 |
| cg23667432 | 0.647 (0.617, 0.671) | 0.304 (0.253, 0.364) | <0.001 |
| cg03188382 | 0.429 (0.407, 0.453) | 0.376 (0.331, 0.428) | <0.001 |
| cg19713851 | 0.484 (0.437, 0.534) | 0.483 (0.410, 0.557) | 0.339 |
| cg27241845 | 0.577 (0.539, 0.607) | 0.224 (0.187, 0.274) | <0.001 |
| cg03329539 | 0.333 (0.307, 0.356) | 0.318 (0.255, 0.406) | <0.001 |
| cg06644428 | 0.244 (0.175, 0.304) | 0.509 (0.375, 0.619) | <0.001 |
| cg05951221 | NA (NA, NA) | 0.535 (0.472, 0.581) | NA |
| cg21566642 | 0.724 (0.692, 0.747) | 0.627 (0.565, 0.670) | <0.001 |
| cg01940273 | 0.528 (0.488, 0.562) | 0.566 (0.488, 0.630) | <0.001 |
| cg13193840 | 0.409 (0.361, 0.453) | 0.272 (0.214, 0.377) | <0.001 |
| cg17024919 | 0.297 (0.241, 0.356) | 0.368 (0.284, 0.458) | <0.001 |
| cg15693572 | 0.471 (0.393, 0.546) | 0.243 (0.198, 0.293) | <0.001 |
| cg23480021 | 0.561 (0.461, 0.662) | 0.649 (0.570, 0.719) | <0.001 |
| cg03274391 | NA (NA, NA) | 0.384 (0.320, 0.451) | NA |
| cg00501876 | 0.544 (0.518, 0.568) | 0.208 (0.179, 0.242) | <0.001 |
| cg18642234 | 0.427 (0.403, 0.451) | 0.481 (0.412, 0.544) | <0.001 |
| cg15417641 | 0.558 (0.495, 0.622) | 0.809 (0.770, 0.841) | <0.001 |
| cg00336149 | 0.366 (0.316, 0.419) | 0.661 (0.608, 0.712) | <0.001 |
| cg21188533 | 0.507 (0.431, 0.582) | 0.718 (0.666, 0.761) | <0.001 |
| cg19859270 | 0.860 (0.844, 0.873) | 0.754 (0.701, 0.794) | <0.001 |
| cg02657160 | 0.792 (0.777, 0.806) | 0.384 (0.329, 0.441) | <0.001 |
| cg25197194 | 0.462 (0.425, 0.500) | 0.653 (0.591, 0.714) | <0.001 |
| cg08202836 | 0.821 (0.780, 0.851) | 0.772 (0.745, 0.794) | <0.001 |
| cg21121843 | 0.230 (0.195, 0.273) | 0.634 (0.566, 0.687) | <0.001 |
| cg19719391 | 0.521 (0.493, 0.549) | 0.342 (0.289, 0.398) | <0.001 |
| cg24556382 | 0.675 (0.631, 0.714) | 0.365 (0.296, 0.445) | <0.001 |
| cg11554391 | 0.155 (0.137, 0.172) | 0.240 (0.152, 0.353) | <0.001 |
| cg17924476 | NA (NA, NA) | 0.559 (0.477, 0.648) | NA |
| cg08606254 | 0.836 (0.814, 0.854) | 0.774 (0.720, 0.810) | <0.001 |
| cg12806681 | 0.841 (0.821, 0.856) | 0.773 (0.730, 0.803) | <0.001 |
| cg03991871 | NA (NA, NA) | 0.618 (0.529, 0.691) | NA |
| cg23916896 | 0.228 (0.190, 0.268) | 0.594 (0.510, 0.663) | <0.001 |
| cg11902777 | 0.087 (0.074, 0.104) | 0.172 (0.123, 0.243) | <0.001 |
| cg01899089 | 0.434 (0.410, 0.457) | 0.342 (0.289, 0.404) | <0.001 |
| cg05575921 | 0.801 (0.733, 0.826) | 0.553 (0.468, 0.632) | <0.001 |
| cg26703534 | 0.642 (0.618, 0.664) | 0.716 (0.677, 0.746) | <0.001 |
| cg01097768 | 0.664 (0.628, 0.694) | 0.472 (0.403, 0.534) | <0.001 |
| cg14817490 | NA (NA, NA) | 0.571 (0.476, 0.653) | NA |
| cg25648203 | 0.731 (0.706, 0.752) | 0.588 (0.510, 0.658) | <0.001 |
| cg21161138 | 0.658 (0.630, 0.680) | 0.448 (0.373, 0.521) | <0.001 |
| cg03604011 | 0.170 (0.149, 0.191) | 0.075 (0.059, 0.098) | <0.001 |
| cg24090911 | 0.667 (0.635, 0.693) | 0.446 (0.375, 0.518) | <0.001 |
| cg13039251 | 0.576 (0.515, 0.636) | 0.784 (0.731, 0.821) | <0.001 |
| cg05673882 | 0.269 (0.227, 0.312) | 0.154 (0.109, 0.226) | <0.001 |
| cg26908328 | 0.118 (0.104, 0.135) | 0.098 (0.085, 0.117) | <0.001 |
| cg16786458 | 0.359 (0.316, 0.404) | 0.415 (0.337, 0.493) | <0.001 |
| cg14580211 | 0.601 (0.560, 0.643) | 0.356 (0.293, 0.430) | <0.001 |
| cg12513616 | 0.397 (0.374, 0.419) | 0.204 (0.171, 0.244) | <0.001 |
| cg01882991 | 0.594 (0.563, 0.616) | 0.309 (0.267, 0.359) | <0.001 |
| cg06126421 | NA (NA, NA) | 0.406 (0.267, 0.556) | NA |
| cg14753356 | 0.330 (0.279, 0.384) | 0.283 (0.175, 0.446) | <0.001 |
| cg24859433 | 0.786 (0.762, 0.806) | 0.359 (0.275, 0.450) | <0.001 |
| cg15342087 | 0.794 (0.772, 0.811) | 0.356 (0.286, 0.435) | <0.001 |
| cg17619755 | 0.506 (0.469, 0.547) | 0.686 (0.616, 0.735) | <0.001 |
| cg10807309 | 0.304 (0.269, 0.337) | 0.641 (0.571, 0.693) | <0.001 |
| cg15474579 | 0.512 (0.481, 0.544) | 0.257 (0.214, 0.306) | <0.001 |
| cg00931843 | NA (NA, NA) | 0.482 (0.420, 0.536) | NA |
| cg00921574 | 0.146 (0.130, 0.165) | 0.088 (0.073, 0.105) | <0.001 |
| cg19717773 | 0.589 (0.544, 0.634) | 0.750 (0.703, 0.792) | <0.001 |
| cg02451831 | 0.755 (0.733, 0.776) | 0.636 (0.584, 0.674) | <0.001 |
| cg08972170 | 0.484 (0.443, 0.531) | 0.743 (0.698, 0.779) | <0.001 |
| cg19089201 | 0.797 (0.776, 0.811) | 0.568 (0.484, 0.644) | <0.001 |
| cg22132788 | NA (NA, NA) | 0.458 (0.396, 0.527) | NA |
| cg04180046 | 0.502 (0.463, 0.549) | 0.255 (0.199, 0.322) | <0.001 |
| cg12803068 | 0.682 (0.607, 0.759) | 0.520 (0.437, 0.606) | <0.001 |
| cg07826859 | NA (NA, NA) | 0.554 (0.479, 0.615) | NA |
| cg03440944 | 0.667 (0.643, 0.689) | 0.547 (0.487, 0.607) | <0.001 |
| cg21322436 | 0.448 (0.425, 0.469) | 0.669 (0.602, 0.716) | <0.001 |
| cg25949550 | 0.136 (0.123, 0.154) | 0.259 (0.184, 0.341) | <0.001 |
| cg11207515 | 0.348 (0.298, 0.397) | 0.220 (0.182, 0.269) | <0.001 |
| cg17372101 | 0.395 (0.365, 0.429) | 0.278 (0.213, 0.365) | <0.001 |
| cg12276019 | 0.246 (0.210, 0.286) | 0.309 (0.125, 0.542) | <0.001 |
| cg24540678 | 0.192 (0.174, 0.213) | 0.371 (0.265, 0.488) | <0.001 |
| cg13518625 | 0.106 (0.084, 0.133) | 0.061 (0.050, 0.075) | <0.001 |
| cg19589396 | 0.655 (0.615, 0.692) | 0.355 (0.263, 0.467) | <0.001 |
| cg25305703 | 0.591 (0.543, 0.636) | 0.652 (0.539, 0.723) | <0.001 |
| cg12075928 | 0.440 (0.386, 0.483) | 0.166 (0.135, 0.201) | <0.001 |
| cg26361535 | 0.687 (0.634, 0.729) | 0.704 (0.661, 0.742) | <0.001 |
| cg13787850 | 0.382 (0.344, 0.419) | 0.161 (0.138, 0.189) | <0.001 |
| cg01692968 | 0.291 (0.260, 0.324) | 0.242 (0.202, 0.291) | <0.001 |
| cg13910681 | 0.138 (0.126, 0.152) | 0.081 (0.069, 0.096) | <0.001 |
| cg22539182 | 0.376 (0.354, 0.397) | 0.480 (0.370, 0.564) | <0.001 |
| cg25953130 | 0.453 (0.401, 0.504) | 0.156 (0.132, 0.190) | <0.001 |
| cg27312979 | 0.371 (0.322, 0.421) | 0.355 (0.270, 0.451) | <0.001 |
| cg25421530 | 0.728 (0.698, 0.754) | 0.750 (0.683, 0.789) | <0.001 |
| cg01744331 | 0.797 (0.779, 0.816) | 0.767 (0.710, 0.810) | <0.001 |
| cg07123182 | NA (NA, NA) | 0.813 (0.757, 0.852) | NA |
| cg16556677 | 0.746 (0.722, 0.767) | 0.672 (0.615, 0.722) | <0.001 |
| cg26963277 | 0.840 (0.823, 0.852) | 0.734 (0.668, 0.780) | <0.001 |
| cg04039799 | 0.340 (0.316, 0.362) | 0.239 (0.192, 0.310) | <0.001 |
| cg09197783 | 0.633 (0.613, 0.654) | 0.773 (0.720, 0.812) | <0.001 |
| cg16611234 | 0.276 (0.244, 0.311) | 0.270 (0.179, 0.418) | 0.815 |
| cg19254163 | 0.581 (0.546, 0.611) | 0.482 (0.410, 0.545) | <0.001 |
| cg21611682 | 0.520 (0.495, 0.545) | 0.552 (0.479, 0.610) | <0.001 |
| cg14624207 | 0.456 (0.433, 0.478) | 0.345 (0.267, 0.447) | <0.001 |
| cg01901332 | 0.585 (0.543, 0.627) | 0.505 (0.435, 0.566) | <0.001 |
| cg11660018 | 0.465 (0.432, 0.496) | 0.493 (0.397, 0.598) | <0.001 |
| cg23771366 | 0.387 (0.354, 0.417) | 0.369 (0.299, 0.461) | <0.001 |
| cg03234777 | 0.140 (0.119, 0.169) | 0.577 (0.499, 0.646) | <0.001 |
| cg26282236 | 0.530 (0.492, 0.570) | 0.745 (0.711, 0.774) | <0.001 |
| cg02583484 | 0.287 (0.257, 0.314) | 0.195 (0.158, 0.252) | <0.001 |
| cg04158018 | 0.251 (0.231, 0.276) | 0.332 (0.246, 0.434) | <0.001 |
| cg23681440 | 0.353 (0.322, 0.387) | 0.214 (0.164, 0.276) | <0.001 |
| cg23126342 | NA (NA, NA) | 0.434 (0.332, 0.558) | NA |
| cg25491122 | 0.751 (0.698, 0.784) | 0.527 (0.450, 0.602) | <0.001 |
| cg06885459 | 0.468 (0.429, 0.504) | 0.551 (0.474, 0.614) | <0.001 |
| cg17487894 | 0.465 (0.440, 0.493) | 0.551 (0.465, 0.617) | <0.001 |
| cg01731783 | 0.608 (0.577, 0.639) | 0.332 (0.288, 0.377) | <0.001 |
| cg22851561 | 0.517 (0.482, 0.551) | 0.459 (0.396, 0.522) | <0.001 |
| cg24996979 | 0.183 (0.167, 0.204) | 0.239 (0.181, 0.319) | <0.001 |
| cg10919522 | 0.215 (0.189, 0.242) | 0.198 (0.159, 0.251) | <0.001 |
| cg13976502 | 0.420 (0.400, 0.439) | 0.423 (0.358, 0.491) | 0.052 |
| cg13038618 | 0.447 (0.417, 0.479) | 0.628 (0.563, 0.683) | <0.001 |
| cg05875421 | 0.145 (0.127, 0.164) | 0.481 (0.397, 0.555) | <0.001 |
| cg05284742 | 0.658 (0.632, 0.688) | 0.789 (0.761, 0.815) | <0.001 |
| cg06819357 | 0.540 (0.513, 0.570) | 0.544 (0.500, 0.585) | 0.338 |
| cg26242531 | 0.321 (0.287, 0.354) | 0.300 (0.248, 0.365) | <0.001 |
| cg11730703 | 0.729 (0.687, 0.762) | 0.707 (0.653, 0.747) | <0.001 |
| cg01208318 | 0.374 (0.312, 0.438) | 0.456 (0.387, 0.523) | <0.001 |
| cg15022400 | 0.158 (0.136, 0.185) | 0.461 (0.385, 0.529) | <0.001 |
| cg03489965 | NA (NA, NA) | 0.694 (0.634, 0.740) | NA |
| cg18335991 | 0.693 (0.669, 0.714) | 0.502 (0.418, 0.590) | <0.001 |
| cg00310412 | 0.459 (0.436, 0.482) | 0.351 (0.302, 0.415) | <0.001 |
| cg11152412 | 0.109 (0.096, 0.123) | 0.105 (0.086, 0.130) | <0.001 |
| cg23161492 | 0.235 (0.206, 0.268) | 0.389 (0.328, 0.442) | <0.001 |
| cg05194346 | 0.433 (0.388, 0.480) | 0.644 (0.588, 0.689) | <0.001 |
| cg01207684 | 0.655 (0.620, 0.690) | 0.350 (0.292, 0.429) | <0.001 |
| cg09099830 | 0.545 (0.510, 0.587) | 0.513 (0.450, 0.569) | <0.001 |
| cg03155159 | 0.400 (0.355, 0.440) | 0.198 (0.159, 0.254) | <0.001 |
| cg00911794 | 0.441 (0.398, 0.470) | 0.466 (0.343, 0.587) | <0.001 |
| cg23621097 | 0.390 (0.340, 0.422) | 0.372 (0.249, 0.507) | 0.238 |
| cg09858022 | 0.414 (0.380, 0.450) | 0.215 (0.185, 0.259) | <0.001 |
| cg19572487 | 0.466 (0.425, 0.502) | 0.333 (0.280, 0.388) | <0.001 |
| cg04956244 | 0.545 (0.527, 0.563) | 0.534 (0.484, 0.577) | <0.001 |
| cg16255816 | 0.307 (0.284, 0.328) | 0.208 (0.173, 0.258) | <0.001 |
| cg03373393 | 0.152 (0.136, 0.168) | 0.252 (0.149, 0.390) | <0.001 |
| cg25809905 | 0.483 (0.446, 0.526) | 0.688 (0.633, 0.736) | <0.001 |
| cg21280392 | 0.386 (0.361, 0.412) | 0.340 (0.285, 0.412) | <0.001 |
| cg07465627 | 0.319 (0.295, 0.344) | 0.630 (0.566, 0.683) | <0.001 |
| cg02186444 | 0.509 (0.487, 0.534) | 0.284 (0.239, 0.331) | <0.001 |
| cg07251887 | 0.398 (0.363, 0.435) | 0.325 (0.274, 0.379) | <0.001 |
| cg06459104 | 0.360 (0.308, 0.413) | 0.347 (0.267, 0.465) | 0.214 |
| cg00073090 | 0.316 (0.294, 0.340) | 0.146 (0.117, 0.184) | <0.001 |
| cg15187398 | 0.432 (0.390, 0.466) | 0.645 (0.593, 0.689) | <0.001 |
| cg07381806 | 0.636 (0.607, 0.663) | 0.568 (0.509, 0.619) | <0.001 |
| cg00835193 | 0.798 (0.767, 0.821) | 0.513 (0.392, 0.633) | <0.001 |
| cg03636183 | 0.588 (0.544, 0.615) | 0.533 (0.464, 0.609) | <0.001 |
| cg15159987 | 0.595 (0.561, 0.626) | 0.679 (0.607, 0.738) | <0.001 |
| cg23973524 | 0.553 (0.519, 0.587) | 0.338 (0.280, 0.394) | <0.001 |
| cg11902728 | 0.645 (0.615, 0.669) | 0.702 (0.649, 0.738) | <0.001 |
| cg22649124 | 0.199 (0.175, 0.221) | 0.194 (0.146, 0.240) | 0.006 |
| cg11701312 | 0.495 (0.476, 0.514) | 0.281 (0.241, 0.327) | <0.001 |
| cg16201146 | 0.656 (0.618, 0.690) | 0.323 (0.244, 0.426) | <0.001 |
| cg07339236 | 0.132 (0.113, 0.159) | 0.291 (0.147, 0.548) | <0.001 |
| cg00871610 | 0.461 (0.429, 0.491) | 0.390 (0.319, 0.466) | <0.001 |
| cg06595162 | 0.680 (0.657, 0.699) | 0.488 (0.428, 0.539) | <0.001 |
| cg23110422 | 0.781 (0.753, 0.801) | 0.358 (0.289, 0.443) | <0.001 |
| cg22635096 | 0.341 (0.303, 0.378) | 0.409 (0.277, 0.531) | <0.001 |
| cg02532700 | 0.182 (0.152, 0.216) | 0.259 (0.197, 0.332) | <0.001 |
| cg01127300 | 0.467 (0.425, 0.509) | 0.135 (0.110, 0.173) | <0.001 |
| **Zhang et al. 2016** | **3.759 (3.229, 4.483)** | **-7.388 (-8.945, -5.816)** | <0.001 |
| cg05575921 | 0.807 (0.730, 0.845) | 0.657 (0.566, 0.739) | <0.001 |
| cg05951221 | NA (NA, NA) | 0.627 (0.557, 0.679) | NA |
| cg02451831 | 0.778 (0.757, 0.798) | 0.710 (0.662, 0.748) | <0.001 |
| cg06126421 | NA (NA, NA) | 0.486 (0.336, 0.634) | NA |
| **Bollepalli et al. 2019** |  |  |  |
| **Never Smoker** | **601 (26.866)** | **476 (20.941)** | <0.001 |
| **Former Smoker** | **970 (43.362)** | **114 (5.015)** |  |
| **Current Smoker** | **666 (29.772)** | **1683 (74.043)** |  |
| cg00006626 | 0.814 (0.798, 0.827) | 0.849 (0.821, 0.868) | <0.001 |
| cg03133799 | 0.784 (0.762, 0.802) | 0.612 (0.537, 0.678) | <0.001 |
| cg06442199 | 0.551 (0.486, 0.613) | 0.857 (0.832, 0.876) | <0.001 |
| cg10006428 | 0.563 (0.516, 0.611) | 0.370 (0.333, 0.408) | <0.001 |
| cg14156792 | 0.503 (0.445, 0.573) | 0.436 (0.379, 0.495) | <0.001 |
| cg17453416 | 0.696 (0.660, 0.728) | 0.515 (0.434, 0.641) | <0.001 |
| cg24629356 | 0.459 (0.384, 0.534) | 0.358 (0.278, 0.440) | <0.001 |
| cg26048448 | 0.129 (0.109, 0.160) | 0.199 (0.152, 0.271) | <0.001 |
| cg00075467 | 0.518 (0.463, 0.571) | 0.522 (0.446, 0.605) | 0.027 |
| cg03936870 | 0.431 (0.391, 0.470) | 0.629 (0.563, 0.686) | <0.001 |
| cg05951221 | NA (NA, NA) | 0.583 (0.534, 0.630) | NA |
| cg07721625 | 0.295 (0.245, 0.345) | 0.375 (0.316, 0.441) | <0.001 |
| cg10362869 | 0.421 (0.389, 0.455) | 0.759 (0.709, 0.797) | <0.001 |
| cg17535283 | 0.881 (0.871, 0.893) | 0.895 (0.885, 0.904) | <0.001 |
| cg21450627 | 0.775 (0.755, 0.793) | 0.851 (0.824, 0.867) | <0.001 |
| cg22587600 | 0.623 (0.573, 0.676) | 0.520 (0.421, 0.622) | <0.001 |
| cg02725398 | 0.306 (0.260, 0.357) | 0.214 (0.169, 0.271) | <0.001 |
| cg21594961 | 0.111 (0.084, 0.374) | 0.427 (0.373, 0.478) | <0.001 |
| cg00379467 | 0.470 (0.440, 0.505) | 0.454 (0.420, 0.489) | <0.001 |
| cg08786370 | 0.827 (0.619, 0.849) | 0.725 (0.607, 0.770) | <0.001 |
| cg26086649 | 0.143 (0.119, 0.175) | 0.452 (0.315, 0.580) | <0.001 |
| cg06677021 | 0.489 (0.445, 0.536) | 0.743 (0.697, 0.777) | <0.001 |
| cg08663909 | 0.580 (0.536, 0.631) | 0.757 (0.695, 0.808) | <0.001 |
| cg14727987 | 0.260 (0.220, 0.304) | 0.172 (0.137, 0.231) | <0.001 |
| cg23576855 | 0.623 (0.440, 0.670) | 0.565 (0.466, 0.655) | <0.001 |
| cg27016106 | 0.285 (0.240, 0.334) | 0.421 (0.344, 0.499) | <0.001 |
| cg00846554 | 0.372 (0.347, 0.399) | 0.376 (0.316, 0.461) | 0.025 |
| cg01555614 | NA (NA, NA) | 0.170 (0.136, 0.234) | NA |
| cg03464017 | 0.732 (0.629, 0.757) | 0.711 (0.643, 0.762) | 0.298 |
| cg06126421 | NA (NA, NA) | 0.531 (0.359, 0.704) | NA |
| cg10525394 | 0.676 (0.629, 0.711) | 0.703 (0.623, 0.764) | <0.001 |
| cg15064086 | 0.415 (0.365, 0.463) | 0.575 (0.496, 0.636) | <0.001 |
| cg18315060 | 0.748 (0.678, 0.795) | 0.775 (0.723, 0.811) | <0.001 |
| cg19643109 | 0.496 (0.441, 0.557) | 0.567 (0.512, 0.630) | <0.001 |
| cg19627238 | 0.648 (0.590, 0.715) | 0.648 (0.554, 0.719) | 0.013 |
| cg26169299 | 0.278 (0.225, 0.320) | 0.577 (0.520, 0.624) | <0.001 |
| cg02243946 | 0.886 (0.869, 0.902) | 0.868 (0.820, 0.891) | <0.001 |
| cg03102898 | 0.584 (0.525, 0.617) | 0.480 (0.430, 0.530) | <0.001 |
| cg26558023 | 0.615 (0.557, 0.676) | 0.751 (0.674, 0.802) | <0.001 |
| cg27650870 | 0.424 (0.391, 0.463) | 0.493 (0.423, 0.557) | <0.001 |
| cg13910813 | NA (NA, NA) | 0.691 (0.549, 0.758) | NA |
| cg06715410 | 0.621 (0.588, 0.654) | 0.874 (0.837, 0.896) | <0.001 |
| cg12589188 | 0.567 (0.503, 0.633) | 0.405 (0.334, 0.494) | <0.001 |
| cg03245590 | 0.192 (0.169, 0.220) | 0.369 (0.268, 0.457) | <0.001 |
| cg05323345 | 0.217 (0.180, 0.264) | 0.330 (0.274, 0.399) | <0.001 |
| cg06120313 | 0.619 (0.404, 0.644) | 0.687 (0.485, 0.755) | <0.001 |
| cg09068031 | 0.750 (0.706, 0.786) | 0.822 (0.782, 0.846) | <0.001 |
| cg05979241 | 0.525 (0.474, 0.578) | 0.440 (0.367, 0.526) | <0.001 |
| cg10609256 | 0.613 (0.551, 0.678) | 0.736 (0.626, 0.800) | <0.001 |
| cg10957001 | 0.841 (0.796, 0.867) | 0.697 (0.611, 0.758) | <0.001 |
| cg16117605 | 0.051 (0.042, 0.063) | 0.155 (0.134, 0.180) | <0.001 |
| cg18161956 | 0.473 (0.434, 0.519) | 0.821 (0.785, 0.846) | <0.001 |
| cg19091257 | 0.420 (0.364, 0.474) | 0.296 (0.249, 0.352) | <0.001 |
| cg21733098 | 0.646 (0.594, 0.696) | 0.833 (0.763, 0.864) | <0.001 |
| cg07499182 | 0.424 (0.343, 0.498) | 0.275 (0.218, 0.371) | <0.001 |
| cg23126342 | NA (NA, NA) | 0.610 (0.491, 0.720) | NA |
| cg23942311 | 0.666 (0.617, 0.807) | 0.509 (0.403, 0.630) | <0.001 |
| cg26103168 | 0.849 (0.835, 0.862) | 0.837 (0.799, 0.866) | <0.001 |
| cg18106898 | 0.471 (0.432, 0.509) | 0.735 (0.693, 0.768) | <0.001 |
| cg01273991 | 0.676 (0.627, 0.728) | 0.776 (0.719, 0.821) | <0.001 |
| cg22947000 | 0.274 (0.243, 0.306) | 0.251 (0.210, 0.294) | <0.001 |
| cg00066239 | 0.719 (0.672, 0.768) | 0.783 (0.709, 0.830) | <0.001 |
| cg02806012 | 0.459 (0.413, 0.502) | 0.337 (0.285, 0.406) | <0.001 |
| cg12438330 | 0.717 (0.654, 0.772) | 0.743 (0.665, 0.807) | <0.001 |
| cg13619177 | 0.784 (0.742, 0.814) | 0.808 (0.756, 0.841) | <0.001 |
| cg18268547 | 0.354 (0.325, 0.386) | 0.850 (0.817, 0.872) | <0.001 |
| cg19572487 | 0.465 (0.428, 0.502) | 0.487 (0.420, 0.558) | <0.001 |
| cg27252019 | NA (NA, NA) | 0.160 (0.133, 0.203) | NA |
| cg27594073 | 0.650 (0.592, 0.713) | 0.566 (0.499, 0.633) | <0.001 |
| cg15129815 | 0.748 (0.426, 0.784) | 0.803 (0.633, 0.859) | <0.001 |
| cg06597652 | 0.470 (0.423, 0.514) | 0.323 (0.251, 0.418) | <0.001 |
| cg08955995 | 0.250 (0.209, 0.287) | 0.194 (0.153, 0.264) | <0.001 |
| cg09298273 | 0.555 (0.510, 0.609) | 0.780 (0.686, 0.839) | <0.001 |
| cg20618441 | 0.135 (0.109, 0.171) | 0.618 (0.561, 0.673) | <0.001 |
| cg22316634 | 0.750 (0.713, 0.775) | 0.817 (0.685, 0.854) | <0.001 |
| cg22331349 | 0.421 (0.391, 0.455) | 0.385 (0.296, 0.535) | <0.001 |
| cg13791092 | 0.372 (0.334, 0.409) | 0.316 (0.274, 0.361) | <0.001 |
| cg26029902 | 0.139 (0.119, 0.169) | 0.368 (0.202, 0.562) | <0.001 |
| cg01103827 | 0.262 (0.233, 0.295) | 0.297 (0.242, 0.355) | <0.001 |
| cg01080924 | 0.164 (0.139, 0.200) | 0.242 (0.209, 0.279) | <0.001 |
| cg10531774 | NA (NA, NA) | 0.866 (0.781, 0.893) | NA |
| cg13451356 | 0.567 (0.534, 0.600) | 0.493 (0.459, 0.526) | <0.001 |
| cg21566642 | 0.519 (0.463, 0.569) | 0.383 (0.288, 0.489) | <0.001 |
| cg24079702 | NA (NA, NA) | 0.063 (0.050, 0.082) | NA |
| cg25242471 | 0.355 (0.299, 0.421) | 0.206 (0.149, 0.278) | <0.001 |
| cg13944838 | 0.672 (0.598, 0.893) | 0.621 (0.396, 0.929) | <0.001 |
| cg16867657 | 0.702 (0.658, 0.744) | 0.790 (0.652, 0.861) | <0.001 |
| cg17619755 | 0.496 (0.452, 0.551) | 0.757 (0.620, 0.841) | <0.001 |
| cg25437304 | 0.523 (0.446, 0.604) | 0.346 (0.251, 0.454) | <0.001 |
| cg26694437 | 0.253 (0.220, 0.297) | 0.434 (0.315, 0.543) | <0.001 |
| cg07179816 | 0.067 (0.039, 0.123) | 0.225 (0.108, 0.382) | <0.001 |
| cg13796381 | 0.397 (0.359, 0.435) | 0.110 (0.079, 0.167) | <0.001 |
| cg02431260 | NA (NA, NA) | 0.165 (0.123, 0.214) | NA |
| cg13771313 | 0.510 (0.445, 0.576) | 0.114 (0.090, 0.150) | <0.001 |
| cg04545296 | 0.160 (0.119, 0.207) | 0.085 (0.069, 0.110) | <0.001 |
| cg16775095 | 0.478 (0.423, 0.748) | 0.232 (0.126, 0.483) | <0.001 |
| cg23766254 | 0.441 (0.382, 0.493) | 0.333 (0.216, 0.529) | <0.001 |
| cg24803719 | 0.325 (0.276, 0.386) | 0.443 (0.370, 0.499) | <0.001 |
| cg16113156 | 0.602 (0.516, 0.696) | 0.594 (0.515, 0.660) | <0.001 |
| cg23513183 | 0.869 (0.824, 0.903) | 0.868 (0.840, 0.889) | 0.014 |
| cg18369516 | 0.491 (0.421, 0.557) | 0.734 (0.528, 0.839) | <0.001 |
| cg25221984 | 0.437 (0.386, 0.493) | 0.510 (0.368, 0.659) | <0.001 |
| cg06644428 | 0.117 (0.089, 0.153) | 0.435 (0.226, 0.602) | <0.001 |
| cg09173768 | 0.392 (0.347, 0.451) | 0.842 (0.775, 0.876) | <0.001 |
| cg03847932 | 0.818 (0.732, 0.918) | 0.351 (0.223, 0.502) | <0.001 |
| cg05575921 | 0.805 (0.734, 0.841) | 0.581 (0.455, 0.721) | <0.001 |
| cg20738735 | 0.873 (0.807, 0.920) | 0.950 (0.942, 0.955) | <0.001 |
| cg24854181 | 0.871 (0.822, 0.903) | 0.683 (0.556, 0.762) | <0.001 |
| cg20839206 | 0.323 (0.291, 0.364) | 0.483 (0.433, 0.531) | <0.001 |
| cg05425699 | 0.514 (0.437, 0.660) | 0.589 (0.497, 0.669) | <0.001 |
| cg00639837 | 0.934 (0.912, 0.949) | 0.512 (0.407, 0.706) | <0.001 |
| cg05293490 | 0.590 (0.523, 0.666) | 0.612 (0.570, 0.651) | <0.001 |
| cg18877361 | 0.723 (0.683, 0.759) | 0.856 (0.742, 0.899) | <0.001 |
| cg13626582 | 0.139 (0.111, 0.207) | 0.713 (0.613, 0.784) | <0.001 |
| cg02267536 | 0.704 (0.674, 0.738) | 0.859 (0.762, 0.896) | <0.001 |
| cg06279276 | 0.306 (0.262, 0.354) | 0.391 (0.172, 0.625) | <0.001 |
| cg06394109 | 0.640 (0.496, 0.809) | 0.289 (0.231, 0.345) | <0.001 |
| cg27436995 | NA (NA, NA) | 0.407 (0.248, 0.588) | NA |
| cg00627029 | 0.332 (0.299, 0.371) | 0.388 (0.300, 0.508) | <0.001 |
| cg00593900 | 0.262 (0.207, 0.320) | 0.062 (0.053, 0.077) | <0.001 |
| cg16702083 | 0.308 (0.171, 0.418) | 0.114 (0.064, 0.265) | <0.001 |


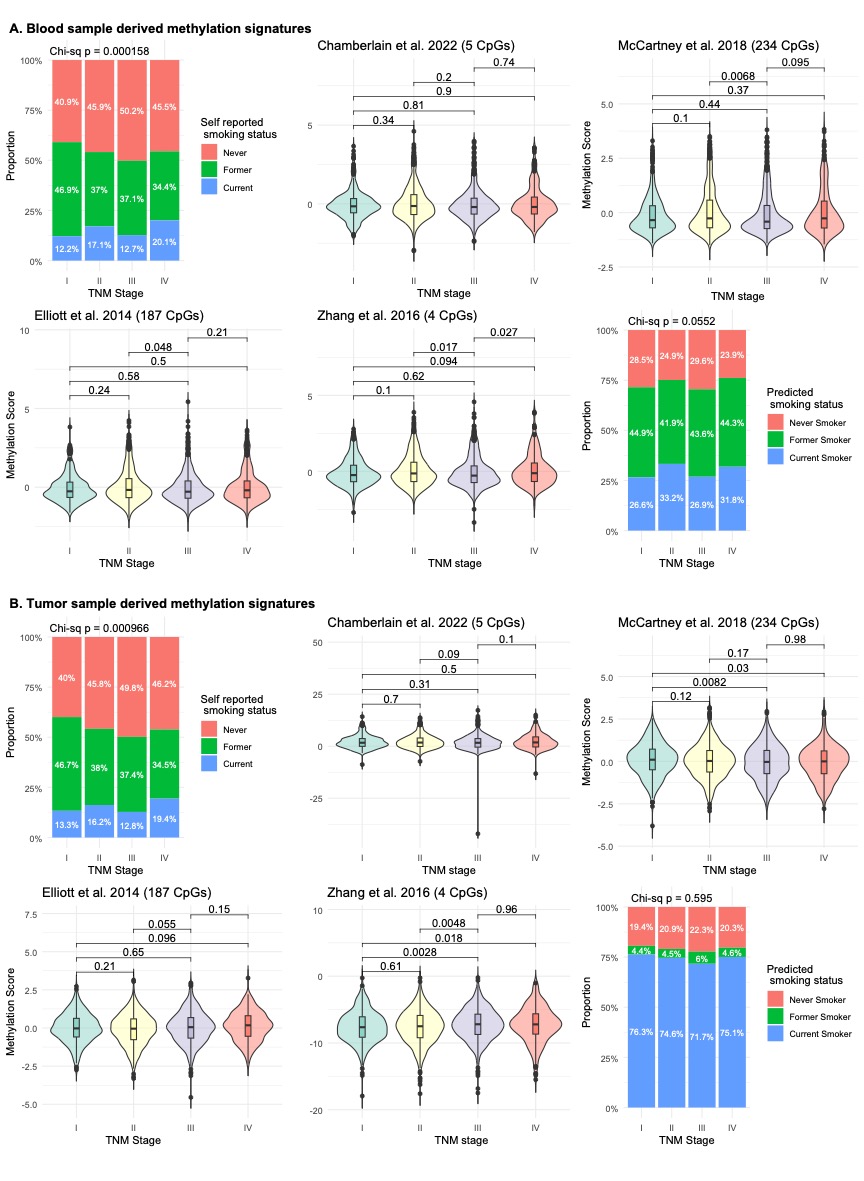


## eFigure1 Distribution of self-reported smoking status and methylation-based smoking signatures/status by TNM stage

## eTable 3. Association between methylation-based smoking scores and self-reported smoking status among patients without treatment with chemotherapy or radiotherapy

|  | **Blood sample (N = 1150)** |  | **Tumor sample (N = 1205)** | |
| --- | --- | --- | --- | --- |
|  | **aOR (95% CI)** | **AUROC (95% CI)** | **aOR (95% CI)** | **AUROC (95% CI)** |
| **Current vs. Never** |  |  |  |  |
| Chamberlainet al. 2022 | 10.42 (6.72, 16.15) | 0.92 (0.9, 0.95) | 1.02 (0.83, 1.25) | 0.48 (0.43, 0.52) |
| McCartney et al. 2018 | 24.71 (13.42, 45.48) | 0.97 (0.95, 0.98) | 1.36 (1.11, 1.67) | 0.56 (0.51, 0.61) |
| Elliott et al. 2014 | 17.81 (10.4, 30.49) | 0.95 (0.93, 0.97) | 1.47 (1.19, 1.82) | 0.59 (0.54, 0.64) |
| Zhang et al. 2016 | 12.56 (7.98, 19.75) | 0.94 (0.91, 0.96) | 1.19 (0.98, 1.45) | 0.54 (0.49, 0.59) |
| Bollepalli et al. 2019 | 686.85 (149.14, 3163.11) | 0.92 (0.89, 0.94) | 1.48 (0.89, 2.45) | 0.56 (0.52, 0.59) |
| **Former vs. Never** |  |  |  |  |
| Chamberlainet al. 2022 | 2.17 (1.75, 2.7) | 0.67 (0.64, 0.7) | 1.05 (0.92, 1.21) | 0.52 (0.49, 0.55) |
| McCartney et al. 2018 | 7.5 (5.43, 10.35) | 0.80 (0.77, 0.82) | 1.3 (1.13, 1.49) | 0.56 (0.53, 0.6) |
| Elliott et al. 2014 | 3.37 (2.63, 4.32) | 0.74 (0.71, 0.77) | 1.26 (1.09, 1.45) | 0.56 (0.52, 0.59) |
| Zhang et al. 2016 | 2.85 (2.27, 3.56) | 0.74 (0.71, 0.77) | 1.11 (0.97, 1.28) | 0.51 (0.48, 0.55) |
| Bollepalli et al. 2019 | 2.47 (1.73, 3.52) | 0.71 (0.68, 0.74) | 1.37 (0.7, 2.68) | 0.54 (0.52, 0.57) |
| **Current vs. Former** |  |  |  |  |
| Chamberlainet al. 2022 | 5.11 (3.74, 6.99) | 0.85 (0.81, 0.89) | 0.94 (0.77, 1.15) | 0.5 (0.45, 0.55) |
| McCartney et al. 2018 | 6.52 (4.67, 9.11) | 0.88 (0.84, 0.91) | 0.97 (0.79, 1.17) | 0.5 (0.45, 0.55) |
| Elliott et al. 2014 | 5.02 (3.72, 6.77) | 0.85 (0.81, 0.89) | 1.06 (0.87, 1.3) | 0.54 (0.49, 0.59) |
| Zhang et al. 2016 | 4.81 (3.58, 6.45) | 0.83 (0.79, 0.87) | 1.06 (0.87, 1.29) | 0.52 (0.47, 0.57) |
| Bollepalli et al. 2019 | 6.76 (3.96, 11.53) | 0.74 (0.71, 0.78) | 0.92 (0.38, 2.22) | 0.51 (0.48, 0.55) |

OR = odds ratio; CI = confidence interval; AUROC = Area under the receiver operating characteristic curve.

##

## eTable 4. Sensitivity analyses for the associations between dichotomized methylation-based scores and mortality among patients with stage I-III CRC

| **Methylation-based smoking scores**  **(High vs. Low)^1^** | **Blood sample (*N* = 1911)** | | **Tumor tissue (*N* = 1959)** | |
| --- | --- | --- | --- | --- |
|  | **aHR (95% CI)** | **a*P*** | **aHR (95% CI)** | **a*P*** |
| **Overall mortality** |  |  |  |  |
| Chamberlainet al. 2022 | 1.13 (0.98, 1.29) | 0.090 | 1.01 (0.88, 1.15) | 0.888 |
| McCartney et al. 2018 | 1.20 (1.05, 1.38) | 0.009 | 1.07 (0.94, 1.22) | 0.305 |
| Elliott et al. 2014 | 1.16 (1.02, 1.34) | 0.029 | 1.11 (0.97, 1.27) | 0.135 |
| Zhang et al. 2016 | 1.27 (1.10, 1.46) | 0.001 | 1.13 (0.99, 1.29) | 0.072 |
| **Non-CRC-related mortality** |  |  |  |  |
| Chamberlainet al. 2022 | 1.17 (0.98, 1.40) | 0.076 | 0.96 (0.81, 1.14) | 0.660 |
| McCartney et al. 2018 | 1.32 (1.10, 1.58) | 0.003 | 1.11 (0.93, 1.32) | 0.239 |
| Elliott et al. 2014 | 1.19 (0.99, 1.42) | 0.062 | 0.92 (0.78, 1.10) | 0.372 |
| Zhang et al. 2016 | 1.33 (1.11, 1.60) | 0.002 | 1.02 (0.86, 1.21) | 0.835 |
| **CRC-specific mortality** |  |  |  |  |
| Chamberlainet al. 2022 | 1.07 (0.86, 1.33) | 0.540 | 1.11 (0.90, 1.38) | 0.316 |
| McCartney et al. 2018 | 1.00 (0.81, 1.25) | 0.965 | 1.03 (0.83, 1.27) | 0.804 |
| Elliott et al. 2014 | 1.11 (0.89, 1.38) | 0.375 | 1.41 (1.13, 1.75) | 0.002 |
| Zhang et al. 2016 | 1.19 (0.95, 1.48) | 0.132 | 1.29 (1.04, 1.60) | 0.019 |

HR = hazards ratio, CI = confidence interval; CRC = colorectal cancer. ^1^ The cut-off points were selected as median values.

The multivariable Cox regression model was adjusted for age, sex, BMI at diagnosis, physical activity alcohol consumption, TNM stage, tumor location, and treatment with chemotherapy or radiotherapy.

## eTable 5. The associations between methylation-based smoking signatures and mortality among patients with stage IV CRC

| **Methylation-based smoking scores** | **Blood sample (*N* = 314)** | | **Tumor tissue (*N* = 325)** | |
| --- | --- | --- | --- | --- |
|  | **aHR (95% CI)** | **a*P*** | **aHR (95% CI)** | **a*P*** |
| **All-cause mortality** |  |  |  |  |
| Chamberlainet al. 2022 | 0.92 (0.81, 1.04) | 0.203 | 0.93 (0.82, 1.06) | 0.286 |
| McCartney et al. 2018 | 0.95 (0.83, 1.07) | 0.382 | 0.90 (0.80, 1.01) | 0.083 |
| Elliott et al. 2014 | 0.97 (0.86, 1.10) | 0.662 | 1.03 (0.92, 1.16) | 0.584 |
| Zhang et al. 2016 | 0.98 (0.86, 1.12) | 0.773 | 0.95 (0.85, 1.07) | 0.394 |
| Bollepalli et al. 2019 |  |  |  |  |
| Former vs. never smoker | 0.82 (0.60, 1.12) | 0.214 | 1.03 (0.57, 1.85) | 0.929 |
| Current vs. never smoker | 0.86 (0.62, 1.20) | 0.372 | 0.77 (0.57, 1.03) | 0.081 |
| **Non-CRC-related mortality** |  |  |  |  |
| Chamberlainet al. 2022 | 0.74 (0.42, 1.31) | 0.301 | 1.07 (0.59, 1.95) | 0.829 |
| McCartney et al. 2018 | 0.87 (0.53, 1.44) | 0.595 | 1.20 (0.70, 2.06) | 0.516 |
| Elliott et al. 2014 | 0.73 (0.42, 1.25) | 0.251 | 1.87 (0.97, 3.62) | 0.061 |
| Zhang et al. 2016 | 1.06 (0.61, 1.83) | 0.846 | 2.46 (1.27, 4.75) | 0.007 |
| Bollepalli et al. 2019 |  |  |  |  |
| Former vs. never smoker | 1.95 (0.40, 9.51) | 0.411 | NA^1^ | 0.999 |
| Current vs. never smoker | 1.27 (0.22, 7.18) | 0.789 | 1.14 (0.24, 5.37) | 0.868 |
| **CRC-specific mortality** |  |  |  |  |
| Chamberlainet al. 2022 | 0.93 (0.81, 1.06) | 0.252 | 0.93 (0.82, 1.06) | 0.272 |
| McCartney et al. 2018 | 0.94 (0.83, 1.08) | 0.383 | 0.89 (0.79, 1.00) | 0.050 |
| Elliott et al. 2014 | 0.99 (0.87, 1.12) | 0.822 | 1.01 (0.89, 1.14) | 0.906 |
| Zhang et al. 2016 | 0.98 (0.86, 1.12) | 0.812 | 0.91 (0.81, 1.03) | 0.130 |
| Bollepalli et al. 2019 |  |  |  |  |
| Former vs. never smoker | 0.77 (0.56, 1.07) | 0.120 | 1.05 (0.58, 1.89) | 0.879 |
| Current vs. never smoker | 0.84 (0.60, 1.18) | 0.317 | 0.75 (0.55, 1.01) | 0.060 |

CRC = colorectal cancer, HR = hazards ratio, CI = confidence interval, CRC = colorectal cancer, NA = not applicable.

The multivariable Cox regression model was adjusted for age, sex, BMI at diagnosis, physical activity alcohol consumption, TNM stage, tumor location, and treatment with chemotherapy or radiotherapy.

^1^ The score predicted only 15 individuals as former smokers, resulting in a proportion or rate of 0.00.

## eTable 6. Interaction effects of methylation-based smoking signatures with sex and age on mortality outcomes

| **Methylation-based signatures** | **Blood sample (N = 1911)** | | | **Tumor tissue (N = 1959)** | | |
| --- | --- | --- | --- | --- | --- | --- |
|  | **All-cause mortality** | **Non-CRC-related mortality** | **Cancer-specific deaths** | **All-cause mortality** | **Non-CRC-related mortality** | **Cancer-specific deaths** |
| **Interaction terms with Sex (Male)** | | | | | | |
| Chamberlainetet al. 2022 | 0.006 | 0.003 | 0.348 | 0.269 | 0.297 | 0.564 |
| McCartney et al. 2018 | 0.031 | 0.051 | 0.240 | 0.075 | 0.194 | 0.314 |
| Elliott et al. 2014 | 0.019 | 0.026 | 0.354 | 0.046 | 0.028 | 0.586 |
| Zhang et al. 2016 | 0.046 | 0.085 | 0.144 | 0.610 | 0.814 | 0.516 |
| Bollepalli et al. 2019^1^ | 0.106 | 0.197 | 0.429 | 0.554 | 0.94 | 0.417 |
| **Interaction terms with Age (≥ 70 years)** | | | |  | | |
| Chamberlainetet al. 2022 | 0.431 | 0.005 | 0.072 | 0.55 | 0.767 | 0.166 |
| McCartney et al. 2018 | 0.95 | 0.096 | 0.174 | 0.297 | 0.962 | 0.193 |
| Elliott et al. 2014 | 0.464 | 0.003 | 0.124 | 0.029 | 0.005 | 0.407 |
| Zhang et al. 2016 | 0.279 | 0.025 | 0.573 | 0.416 | 0.491 | 0.523 |
| Bollepalli et al. 2019^1^ | 0.306 | 0.026 | 0.533 | 0.883 | 0.843 | 0.878 |

CRC = colorectal cancer, HR = hazards ratio, CI = confidence interval.

Values in the cells are the adjusted P values of interaction terms in multivariable Cox regression models, which were adjusted for age, sex, BMI at diagnosis, physical activity, alcohol consumption, TNM stage, tumor location, and treatment with chemotherapy or radiotherapy. Two interaction terms, Age (≥70 years) *methylation signature and Sex*methylation signature, were included in models. ^1^Predicted current smokers versus never smokers

## eTable 7. Stratified analyses for methylation-based signatures showing significant interaction effect with age or sex

| **Methylation-based signatures** | **Smple source** | **Group** | **aHR (95% CI)** | **a*P* value** |
| --- | --- | --- | --- | --- |
| **All-cause mortality** | | | | |
| Chamberlainetet al. 2022 | Blood | Male | 1.17 (1.07, 1.28) | 0.001 |
|  |  | Female | 0.97 (0.84, 1.12) | 0.702 |
| McCartney et al. 2018 | Blood | Male | 1.21 (1.11, 1.32) | <0.001 |
|  |  | Female | 1.08 (0.94, 1.23) | 0.304 |
| Zhang et al. 2016 | Blood | Male | 1.22 (1.11, 1.33) | <0.001 |
|  |  | Female | 1.07 (0.93, 1.22) | 0.339 |
| Elliott et al. 2014 | Blood | Male | 1.22 (1.12, 1.33) | <0.001 |
|  |  | Female | 1.10 (0.97, 1.26) | 0.141 |
|  | Tumor | Male | 1.11 (1.01, 1.21) | 0.025 |
|  |  | Female | 1.01 (0.90, 1.13) | 0.846 |
|  |  | Age < 70 years | 1.17 (1.04, 1.32) | 0.011 |
|  |  | Age ≥ 70 years | 1.01 (0.93, 1.10) | 0.852 |
| **Non-CRC-related mortality** | | | | |
| Chamberlainetet al. 2022 | Blood | Male | 1.22 (1.09, 1.36) | 0.001 |
|  |  | Female | 0.96 (0.78, 1.17) | 0.666 |
| McCartney et al. 2018 | Blood | Male | 1.27 (1.14, 1.42) | <0.001 |
|  |  | Female | 1.16 (0.95, 1.41) | 0.147 |
| Elliott et al. 2014 | Blood | Male | 1.24 (1.11, 1.39) | <0.001 |
|  |  | Female | 1.12 (0.92, 1.35) | 0.255 |
| Zhang et al. 2016 | Blood | Male | 1.30 (1.16, 1.45) | <0.001 |
|  |  | Female | 1.18 (0.98, 1.41) | 0.080 |
| Chamberlainetet al. 2022 | Blood | Age < 70 years | 1.30 (1.11, 1.52) | 0.001 |
|  |  | Age ≥ 70 years | 1.04 (0.91, 1.18) | 0.585 |
| Zhang et al. 2016 | Blood | Age < 70 years | 1.32 (1.13, 1.54) | 0.001 |
|  |  | Age ≥ 70 years | 1.14 (1.01, 1.30) | 0.036 |
| Bollepalli et al. 2019^1^ | Blood | Age < 70 years | 1.88 (1.23, 2.87) | 0.003 |
|  |  | Age ≥ 70 years | 1.26 (0.94, 1.69) | 0.115 |
| Elliott et al. 2014 | Blood | Age < 70 years | 1.45 (1.25, 1.69) | <0.001 |
|  |  | Age ≥ 70 years | 1.13 (0.99, 1.28) | 0.063 |
|  | Tumor | Age < 70 years | 1.17 (0.98, 1.39) | 0.084 |
|  |  | Age ≥ 70 years | 0.91 (0.82, 1.01) | 0.074 |

CRC = colorectal cancer, HR = hazards ratio, CI = confidence interval.

^1^Predicted current smokers versus never smokers

## eTable 8. Associations between methylation-based smoking scores and self-reported smoking status among overlapping patients with both blood and tumor sample

| **Methylation score** | **Blood sample (N = 2008)** | | **Tumor sample (N = 2008)** | |
| --- | --- | --- | --- | --- |
|  | **aOR (95% CI)** | **AUROC (95% CI)** | **aOR (95% CI)** | **AUROC (95% CI)** |
| **Current vs. Never** |  |  |  |  |
| Chamberlainet al. 2022 | 10.5 (7.65, 14.42) | 0.92 (0.90, 0.94) | 1.08 (0.93, 1.25) | 0.53 (0.50, 0.57) |
| McCartney et al. 2018 | 27.89 (17.53, 44.36) | 0.96 (0.94, 0.97) | 1.46 (1.26, 1.7) | 0.59 (0.55, 0.63) |
| Elliott et al. 2014 | 19.53 (13.01, 29.33) | 0.94 (0.92, 0.96) | 1.45 (1.25, 1.68) | 0.61 (0.58, 0.63) |
| Zhang et al. 2016 | 13.68 (9.76, 19.19) | 0.94 (0.92, 0.95) | 1.3 (1.12, 1.51) | 0.58 (0.54, 0.62) |
| Bollepalli et al. 2019 | 343.29 (144.94, 813.09) | 0.92 (0.90, 0.94) | 1.6 (1.09, 2.35) | 0.55 (0.53, 0.58) |
| **Former vs. Never** |  |  |  |  |
| Chamberlainet al. 2022 | 2.39 (2.02, 2.83) | 0.66 (0.64, 0.69) | 1.06 (0.95, 1.18) | 0.52 (0.50, 0.55) |
| McCartney et al. 2018 | 9.26 (7.09, 12.08) | 0.80 (0.78, 0.82) | 1.17 (1.05, 1.3) | 0.54 (0.51, 0.56) |
| Elliott et al. 2014 | 4.02 (3.29, 4.91) | 0.74 (0.72, 0.76) | 1.26 (1.13, 1.41) | 0.56 (0.53, 0.59) |
| Zhang et al. 2016 | 3.13 (2.62, 3.74) | 0.74 (0.72, 0.76) | 1.13 (1.01, 1.25) | 0.53 (0.50, 0.55) |
| Bollepalli et al. 2019 | 2.1 (1.62, 2.72) | 0.71 (0.69, 0.73) | 1.05 (0.61, 1.81) | 0.54 (0.52, 0.56) |
| **Current vs. Former** |  |  |  |  |
| Chamberlainet al. 2022 | 4.46 (3.6, 5.52) | 0.84 (0.82, 0.87) | 1.00 (0.86, 1.17) | 0.51 (0.47, 0.55) |
| McCartney et al. 2018 | 5.57 (4.44, 6.97) | 0.87 (0.84, 0.89) | 1.12 (0.97, 1.29) | 0.55 (0.52, 0.59) |
| Elliott et al. 2014 | 4.54 (3.69, 5.6) | 0.84 (0.81, 0.86) | 1.13 (0.97, 1.31) | 0.56 (0.52, 0.60) |
| Zhang et al. 2016 | 4.51 (3.65, 5.58) | 0.82 (0.79, 0.85) | 1.1 (0.95, 1.28) | 0.55 (0.51, 0.59) |
| Bollepalli et al. 2019 | 6.18 (4.15, 9.19) | 0.74 (0.71, 0.76) | 0.85 (0.43, 1.71) | 0.51 (0.49, 0.54) |

OR = odds ratio; CI = confidence interval; AUROC = Area under the receiver operating characteristic curve. The multivariable logistic model was adjusted for age, sex, body mass index 5-14 years earlier, alcohol consumption, physical activity, use of nonsteroidal anti-inflammatory drugs, hormone replacement therapy, and prior large bowel endoscopy

## eTable 9. Associations between methylation-based smoking scores and CRC mortality among overlapping stage I-III patients with both blood and tumor methylation data

| **Methylation-based smoking scores** | **Outcomes** | **Blood sample**  **(N = 1718)** | **Tumor sample**  **(N = 1717)** |
| --- | --- | --- | --- |
|  |  | **aHR (95% CI)** | **aHR (95% CI)** |
| Chamberlainet al. 2022 | OS | 1.11 (1.02, 1.20) | 1.02 (0.95, 1.09) |
|  | NCR | 1.16 (1.04, 1.28) | 1.01 (0.93, 1.11) |
|  | CSS | 1.05 (0.92, 1.19) | 1.04 (0.93, 1.16) |
| McCartney et al. 2018 | OS | 1.20 (1.11, 1.29) | 0.97 (0.90, 1.04) |
|  | NCR | 1.29 (1.17, 1.42) | 0.99 (0.91, 1.08) |
|  | CSS | 1.08 (0.96, 1.22) | 0.95 (0.85, 1.06) |
| Elliott et al. 2014 | OS | 1.18 (1.10, 1.28) | 1.02 (0.95, 1.09) |
|  | NCR | 1.26 (1.14, 1.39) | 0.93 (0.85, 1.02) |
|  | CSS | 1.08 (0.96, 1.22) | 1.16 (1.03, 1.30) |
| Zhang et al. 2016 | OS | 1.20 (1.11, 1.30) | 1.05 (0.98, 1.13) |
|  | NCR | 1.25 (1.13, 1.39) | 1.00 (0.91, 1.09) |
|  | CSS | 1.13 (1.00, 1.28) | 1.14 (1.02, 1.28) |
| Bollepalli et al. 2019 | OS |  |  |
| Never smoker |  | 1 | 1 |
| Former Smoker |  | 0.94 (0.79, 1.12) | 0.97 (0.68, 1.38) |
| Current Smoker |  | 1.32 (1.09, 1.60) | 1.04 (0.88, 1.24) |
| Bollepalli et al. 2019 | NCR |  |  |
| Never smoker |  | 1 | 1 |
| Former Smoker |  | 0.93 (0.74, 1.17) | 1.08 (0.69, 1.68) |
| Current Smoker |  | 1.48 (1.16, 1.90) | 1.02 (0.82, 1.27) |
| Bollepalli et al. 2019 | CSS |  |  |
| Never smoker |  | 1 | 1 |
| Former Smoker |  | 1.00 (0.75, 1.32) | 0.80 (0.43, 1.50) |
| Current Smoker |  | 1.07 (0.78, 1.47) | 1.09 (0.83, 1.45) |

CRC = colorectal cancer, OS = overall survival, NCR = non-CRC-related survival, CSS = cancer-specific survival, HR = hazards ratio.

## eTable 10. Associations between methylation-based smoking scores derived from adjacent normal tissue and self-reported smoking status

| **Methylation score** | **AUROC** | **Univariable logistic model** | | | **Multivariable logistic model^1^** | |
| --- | --- | --- | --- | --- | --- | --- |
|  |  | **cOR (95% CI)** | **c*P* value** | **McFadden R^2^** | **aOR (95 CI)** | **a*P* value** |
| **Adjacent normal tissue ( N = 264)** | | | | | | |
| **Current vs. Never** |  |  |  |  |  |  |
| Chamberlainetet al. 2022 | 0.47 | 0.98 (0.86, 1.11) | 0.760 | 0.001 | 0.95 (0.68, 1.33) | 0.765 |
| McCartney et al. 2018 | 0.59 | 1.16 (0.65, 2.07) | 0.613 | 0.001 | 1.11 (0.76, 1.62) | 0.581 |
| Elliott et al. 2014 | 0.51 | 0.97 (0.84, 1.11) | 0.620 | 0.001 | 0.93 (0.63, 1.39) | 0.74 |
| Zhang et al. 2016 | 0.53 | 1.08 (0.88, 1.32) | 0.464 | 0.003 | 1.10 (0.75, 1.63) | 0.624 |
| Bollepalli et al. 2019^2^ | 0.47 | NA^3^ | 0.986 | 0.017 | NA^3^ | 0.991 |
| **Former vs. Never** |  |  |  |  |  |  |
| Chamberlainetet al. 2022 | 0.50 | 1.01 (0.89, 1.15) | 0.825 | <0.001 | 0.97 (0.69, 1.36) | 0.857 |
| McCartney et al. 2018 | 0.50 | 1.06 (0.65, 1.73) | 0.822 | 0.001 | 1.05 (0.76, 1.45) | 0.768 |
| Elliott et al. 2014 | 0.60 | 1.12 (0.99, 1.25) | 0.070 | 0.011 | 1.39 (1.01, 1.92) | 0.046 |
| Zhang et al. 2016 | 0.56 | 1.11 (0.94, 1.31) | 0.204 | 0.005 | 1.27 (0.93, 1.73) | 0.127 |
| Bollepalli et al. 2019^2^ | 0.51 | NA^3^ | 0.988 | 0.012 | NA^3^ | 0.988 |
| **Current vs. Former** |  |  |  |  |  |  |
| Chamberlainetet al. 2022 | 0.47 | 0.97 (0.84, 1.11) | 0.637 | 0.001 | 0.93 (0.62, 1.38) | 0.703 |
| McCartney et al. 2018 | 0.58 | 1.10 (0.64, 1.89) | 0.737 | 0.001 | 1.07 (0.72, 1.60) | 0.746 |
| Elliott et al. 2014 | 0.57 | 0.90 (0.80, 1.03) | 0.122 | 0.014 | 0.64 (0.41, 0.98) | 0.040 |
| Zhang et al. 2016 | 0.53 | 0.98 (0.80, 1.21) | 0.876 | 0.001 | 0.99 (0.62, 1.56) | 0.958 |
| Bollepalli et al. 2019^2^ | 0.46 | 0.80 (0.14, 4.45) | 0.796 | 0.008 | 0.64 (0.19, 2.13) | 0.466 |

OR = odds ratio; CI = confidence interval; AUROC = area under the receiver operating characteristic curves; NA = not applicable. McFadden R^2^ measured the explained variation by blood methylation-based smoking panels.

^1^ The model was adjusted for age, sex, body mass index 5-14 years earlier, alcohol consumption, physical activity, use of nonsteroidal anti-inflammatory drugs, hormone replacement therapy, and prior large bowel endoscopy. ^2^ The association between predicted binary smoking status (e.g., predicted current vs. never smokers) and corresponding self-reported binary outcomes was assessed. ^3^ The classifier predicted only three individuals as never smokers, resulting in a proportion or rate of 0.00.

## eTable 11. Associations between methylation-based smoking scores derived from adjacent normal tissue and mortality

| **Methylation-based smoking scores** | **Stage I-III (N = 231)** | |
| --- | --- | --- |
|  | **aHR (95% CI)** | **a*P*** |
| **All-cause mortality** |  |  |
| Chamberlainet al. 2022 | 1.14 (0.92, 1.41) | 0.232 |
| McCartney et al. 2018 | 1.23 (1.00, 1.52) | 0.055 |
| Elliott et al. 2014 | 1.13 (0.91, 1.41) | 0.259 |
| Zhang et al. 2016 | 0.99 (0.81, 1.23) | 0.962 |
| Bollepalli et al. 2019 |  |  |
| Former vs. never smoker | 2.07 (0.26, 16.51) | 0.492 |
| Current vs. never smoker | 2.06 (0.28, 15.43) | 0.480 |
| **Non-CRC-related mortality** |  |  |
| Chamberlainet al. 2022 | 1.11 (0.86, 1.42) | 0.427 |
| McCartney et al. 2018 | 1.19 (0.92, 1.53) | 0.196 |
| Elliott et al. 2014 | 1.05 (0.81, 1.36) | 0.726 |
| Zhang et al. 2016 | 1.08 (0.84, 1.39) | 0.569 |
| Bollepalli et al. 2019 |  |  |
| Former vs. never smoker | 1.46 (0.17, 12.36) | 0.731 |
| Current vs. never smoker | 1.64 (0.21, 12.73) | 0.634 |
| **CRC-specific mortality** |  |  |
| Chamberlainet al. 2022 | 1.23 (0.81, 1.87) | 0.323 |
| McCartney et al. 2018 | 1.31 (0.91, 1.88) | 0.142 |
| Elliott et al. 2014 | 1.32 (0.87, 2.00) | 0.196 |
| Zhang et al. 2016 | 0.84 (0.57, 1.24) | 0.374 |
| Bollepalli et al. 2019 |  |  |
| Former vs. never smoker | NA^1^ | 0.997 |
| Current vs. never smoker | NA^1^ | 0.997 |

CRC = colorectal cancer, HR = hazards ratio, NA = not applicable.

The multivariable Cox regression model was adjusted for age, sex, BMI at diagnosis, physical activity, alcohol consumption, TNM stage, tumor location, and treatment with chemotherapy or radiotherapy.

^1^ The score predicted only 31 individuals as former smokers, and 2 patients as never smokers, resulting in a proportion or rate of 0.00.
